# Arteries are formed by vein-derived endothelial tip cells

**DOI:** 10.1038/ncomms6758

**Published:** 2014-12-15

**Authors:** Cong Xu, Sana S. Hasan, Inga Schmidt, Susana F. Rocha, Mara E. Pitulescu, Jeroen Bussmann, Dana Meyen, Erez Raz, Ralf H. Adams, Arndt F. Siekmann

**Affiliations:** 1 Max Planck Institute for Molecular Biomedicine, Roentgenstr. 20, 48149 Muenster, Germany; 2 Institute of Cell Biology, ZMBE, Von-Esmarch-Str. 56, 48149 Muenster, Germany; 3 University of Münster, Faculty of Medicine, D-48149 Münster, Germany

## Abstract

Tissue vascularization entails the formation of a blood vessel plexus, which remodels into arteries and veins. Here we show, by using time-lapse imaging of zebrafish fin regeneration and genetic lineage tracing of endothelial cells in the mouse retina, that vein-derived endothelial tip cells contribute to emerging arteries. Our movies uncover that arterial-fated tip cells change migration direction and migrate backwards within the expanding vascular plexus. This behaviour critically depends on chemokine receptor *cxcr4a* function. We show that the relevant Cxcr4a ligand Cxcl12a selectively accumulates in newly forming bone tissue even when ubiquitously overexpressed, pointing towards a tissue-intrinsic mode of chemokine gradient formation. Furthermore, we find that *cxcr4a* mutant cells can contribute to developing arteries when in association with wild-type cells, suggesting collective migration of endothelial cells. Together, our findings reveal specific cell migratory behaviours in the developing blood vessel plexus and uncover a conserved mode of artery formation.

## Abstract (web summary)

Sprouting of new blood vessels depends on the migration of endothelial tip cells into surrounding tissue. Here the authors reveal the existence of a distinct migratory signalling circuit that guides endothelial cells from developing veins to the leading tip position in developing arteries.

## Graphical abstract

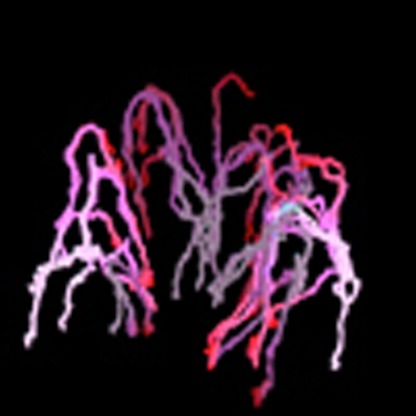

The formation of new blood vessels is a crucial process during embryogenesis and growth^1^,^2^,^3^,^4^, but also in regenerative processes, such as wound healing and tissue repair^5^. It entails the tight coordination of different cellular processes, such as proliferation, migration and lumen formation. In the early embryo, vascular development is often stereotypical, resulting in the formation of identical looking vascular networks^6^. By contrast, at later stages many vascular beds form via a plexus intermediate, which remodels into a hierarchical network of arteries and veins. Examples are the emerging skin blood vessels^7^ or the postnatally forming blood vessels of the mouse retina^8^. In addition, the vasculature in regenerating tissues, for instance in the regenerating zebrafish fin^9^ or during wound healing^10^, forms via a plexus.

In the current concepts of blood vessel formation, an initial pro-angiogenic cue specifies endothelial tip cells, which become motile and navigate through the avascular tissue^11^. Tip cells are followed by stalk cells. These maintain the connection to the pre-existing vasculature, are less motile and subsequently establish a hierarchical network of arteries and veins to allow for efficient tissue perfusion^12^. Further studies have investigated the dynamics of endothelial cell migration during sprout outgrowth. Jakobsson *et al.*^13^ showed in an embryoid body sprouting assay that endothelial cells compete for the tip cell position. Another study using a mouse aortic ring assay suggested that this competition and the ensuing ‘cell-mixing’ are important for the proper outgrowth of angiogenic sprouts^14^.

Despite the insights these studies provided, they were performed in *in vitro* settings, which lack proper arterial-venous differentiation of the forming vascular plexus and tissue perfusion. So far, *in vivo* imaging of the forming vasculature has been mainly performed in transparent zebrafish embryos^15^,^16^,^17^. However, these studies have not included vascular beds that form via a plexus intermediate. Therefore, despite the significance of vascular plexus formation and subsequent remodelling for tissue perfusion, we still have a poor understanding of endothelial cell dynamics during these processes. In particular, we do not understand how endothelial cells coordinate the sprouting of new vessels with the establishment of larger arteries and veins.

In this study, we take advantage of the optical clarity of the adult zebrafish fin to perform *in vivo* time-lapse imaging of the complex cell migratory behaviours during blood vessel formation in regenerating tissues. Our results show that endothelial tip cells not only invade avascular tissues, but that they can subsequently change their direction of migration and ultimately migrate against the advancing vascular front. Furthermore, we show that this behaviour is necessary for the proper formation of arteries. In addition, we observe proliferating endothelial cells in tip and stalk cells of venous origin, while arterial endothelial cells proliferate less. Through genetic lineage tracing in the mouse retina, we provide evidence that this mode of artery formation is conserved in other vascular beds that form via a plexus intermediate. We implicate the chemokine receptor Cxcr4a in regulating these migratory behaviours and suggest that a tissue-intrinsic mode is responsible for the generation of a Cxcl12a chemokine gradient. We finally demonstrate through cell transplantation experiments that endothelial cells show a collective cell migration behaviour.

## Results

### Blood vessels regenerate via an intermediate vascular plexus

**Figure 1:**
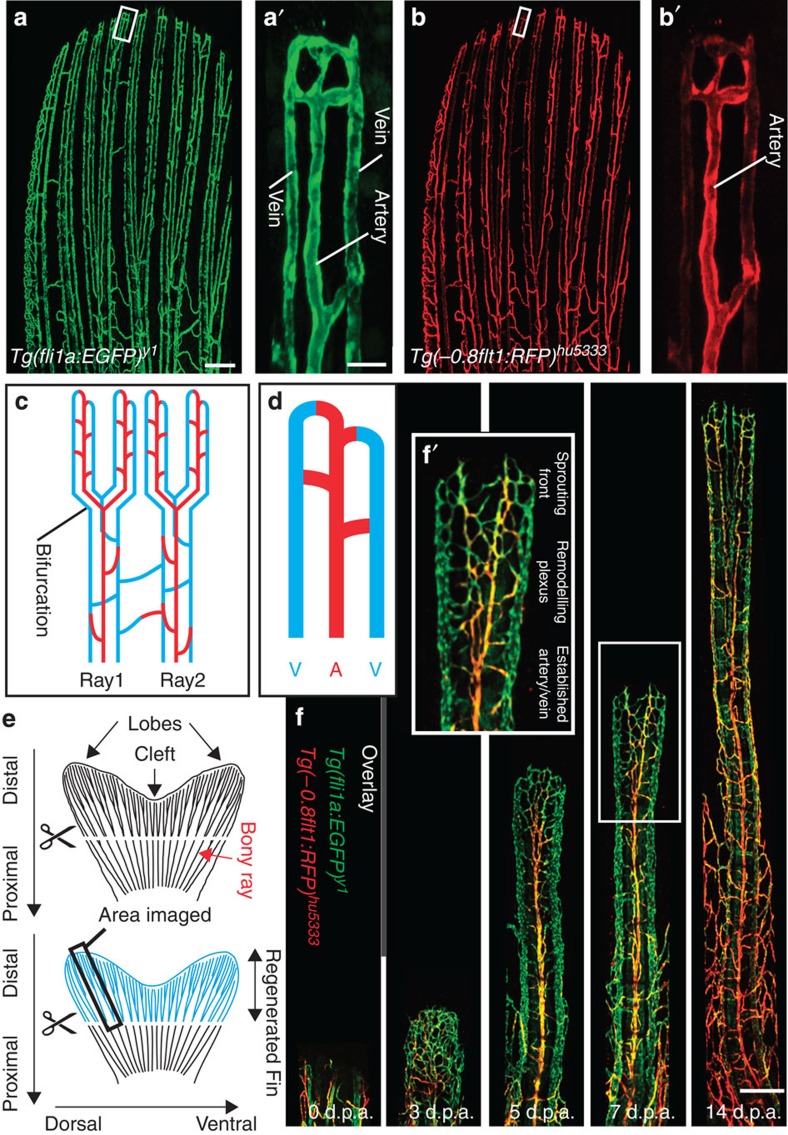
Visualizing blood vessels in the regenerating zebrafish fin. (**a**,**a**′) *Tg(fli1a:EGFP)*^*y1*^ labels all fin blood vessels. (**b**,**b**′) *Tg(−0.8flt1:RFP)*^*hu5333*^ preferentially labels arterial endothelial cells. (**c**,**d**) Schematic drawings of the zebrafish fin vasculature, indicating the ray bifurcation, arteries (A) and veins (V). (**e**) Schematic of zebrafish fin, indicating plane of amputation and imaged area. (**f**) Overlay of *Tg(fli1a:EGFP)*^*y1*^ and *Tg(−0.8flt1:RFP)*^*hu5333*^ at different stages of fin regeneration highlighting the medially located arterial cells in yellow. Inset (**f**′) shows remodelling vascular plexus, which is located proximal to the sprouting front and distally to the established arteries and veins. Scale bar in **a** is 80 μm, in **a**′ is 20 μm and 200 μm in **f**. Representative images from a total of three zebrafish imaged per stage.

To visualize regenerating blood vessels, we analysed vascular dynamics during fin regeneration in adult transgenic zebrafish. In *Tg(fli1a:EGFP)*^*y1*^ zebrafish, all endothelial cells are labelled by EGFP expression^18^, while, in *Tg(−0.8flt1:RFP)*^*hu5333*^ fish, arterial endothelial cells are labelled by high RFP expression^19^. Confocal imaging of fins of double transgenic animals revealed that, in the distal part, each fin ray contained a medially located artery, which was flanked by two veins (Fig. 1a–d). These vessels furthermore showed distinct topologies in respect to the bones of the fin rays. While the artery ran within the bone, the veins were located outside of the bony rays (Supplementary Fig. 1a–g). Thus, the vasculature of the zebrafish fin consists of a regular pattern of arteries and veins, which can be visualized using different transgenic zebrafish lines.

We next amputated the fins of double transgenic animals and observed blood vessel growth within the regenerating tissue (Fig. 1e). In agreement with previous observations^9^, we detected the formation of a dense vascular plexus at 3 days post amputation (Fig. 1f, 3 d.p.a.). This plexus, after bifurcating, subsequently remodelled into a single medial artery and two lateral veins (Fig. 1f, 5–14 d.p.a., see also Supplementary Fig. 2). We could distinguish different areas within the vasculature distally to the established artery and vein during this outgrowth phase. Most distally, we observed a sprouting front invading the avascular tissue, followed by a remodelling vascular plexus (Fig. 1f’). Within this plexus, we could distinguish arterial fated endothelial cells by virtue of RFP expression (see also Supplementary Fig. 2c). Therefore, our double transgenic zebrafish allowed for the detailed observation of the forming vasculature via a plexus intermediate during tissue regeneration.

### Time-lapse imaging reveals vein cells contribute to arteries

**Figure 2:**
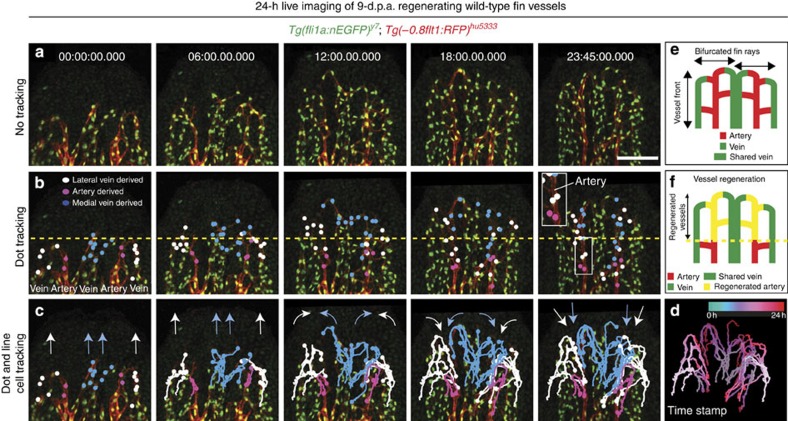
Time-lapse imaging of regenerating blood vessels reveals contribution of vein-derived tip cells to the forming artery. (**a**) Still images taken every 6 h from a 24-h time-lapse movie on 9 d.p.a. fin regenerate of wild-type fish. (**b**) Tracking of individual cells deriving form the lateral vein (white dots), the artery (pink dots) or the medial vein (blue dots). While arterial cells hardly contribute to the advancing front, vein-derived cells contribute to the newly forming artery (inset at 23:45 h time point). (**c**) Tracks of labelled cells in (**b**). White arrows indicate migration paths of lateral vein-derived endothelial cells, while blue arrows indicate migration paths of medial vein-derived endothelial cells. Note change in migration direction. (**d**) Time stamp of tracked endothelial cells. (**e**) Schematic drawing of vessel front in the regenerated fin of wild-type fish at 10 d.p.a. Arteries and veins are indicated. (**f**) Schematic drawing of wild-type fin vasculature regenerated within 24 h from 9 d.p.a. Scale bar, 100 μm; d.p.a.=days post amputation. Representative movie of a total of three movies is shown.

To detect endothelial cell movements, and arterial differentiation, we combined *Tg(fli1a:nEGFP)*^*y7*^, labelling endothelial cell nuclei^20^ with *Tg(−0.8flt1:RFP)*^*hu5333*^ fish and performed time-lapse imaging for 24 h, starting at 9 d.p.a. (Supplementary Movie 1). Representative images taken every 6 h can be seen in Fig. 2. At this stage, the fin ray started to bifurcate and consisted of two arteries flanked by a shared vein in the middle and two lateral veins. We then colour coded endothelial cells located in arterial and venous positions and followed their migratory paths (Fig. 2b,c). This analysis revealed prominent differences in migratory behaviours between venous- and arterial-derived endothelial cells during tissue vascularization. Cells located in venous positions migrated more extensively towards the sprouting front than arterial-derived endothelial cells (compare blue/white dots and tracks with pink ones in Fig. 2b,c). Furthermore, some of these vein-derived endothelial tip cells subsequently changed their orientation, turned medially and finally migrated against the direction of the moving vascular front (arrows in Fig. 2c, time stamp in d, schematic drawings in e, f). Strikingly, we observed that the cells that had turned medially formed the remodelling artery in the centre of each fin ray (Fig. 2b, 23:45 h time point, inset). By contrast, we did not observe endothelial cells coming from the artery in venous blood vessels. We furthermore quantified proliferation of vein- and artery-derived endothelial cells (Supplementary Fig. 3a,b; Supplementary Movie 2). Whereas about 50% of venous endothelial cells divided within 24 h, less than 20% of artery-derived endothelial cells proliferated (Supplementary Fig. 3e). We detected both dividing tip- and stalk cells (Supplementary Fig. 4 and Supplementary Movie 3). Thus, our time-lapse analysis of blood vessel growth reveals a venous origin of arterial endothelial cells and shows that distinct migratory properties of endothelial tip cells contribute to proper blood vessel morphogenesis during tissue regeneration. It also shows that pronounced differences exist in endothelial cell proliferation between arteries and veins.

### Tip cells in the mouse retinal plexus contribute to arteries

**Figure 3:**
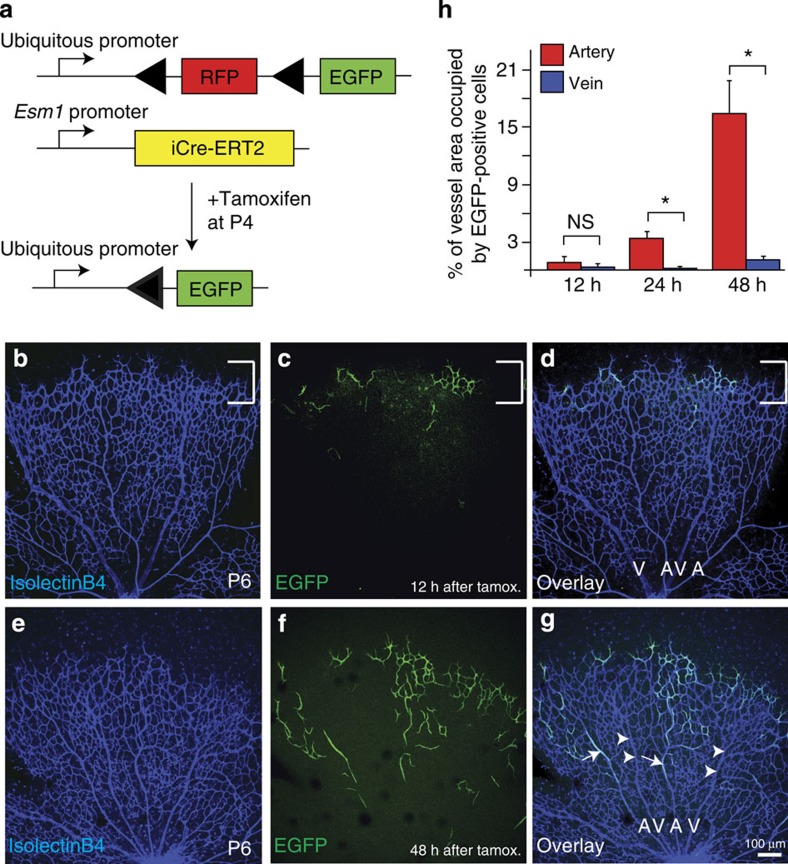
Genetic lineage tracing of tip cells in the mouse retinal vascular plexus. (**a**) Schematic of genetic lineage tracing and transgenic lines used. (**b**) Isolectin B4 staining of the retinal vasculature at P6. Bracket indicates vascular front. (**c**) Location of EGFP expressing cells at the vascular front 12 h after tamoxifen administration. (**d**) Overlay of isolectin B4 and EGFP channels. Remodelling arteries (A) and veins (V) are marked. (**e**) Isolectin B4 staining of the retinal vasculature at P6. (**f**) Location of EGFP expressing cells at the vascular front 48 h after tamoxifen administration. (**g**) Overlay of isolectin B4 and EGFP channels. Remodelling arteries (A) and veins (V) are marked. Arrows indicate EGFP-positive endothelial cells in arteries, while arrowheads indicate EGFP-negative veins. (**h**) Percentage of EGFP-expressing endothelial cells in arteries and veins at different time points after tamoxifen administration. Scale bar, 100 μm. For each time point five mice were analysed. *P<0.05; *t*-test, error bars indicate s.e.m.

We next wanted to know whether blood vessel formation in other vascular beds that are established via a plexus intermediate occurs by a similar mechanism. We chose to investigate the mouse retinal vasculature due to the availability of genetic tools that allow for lineage tracing of different cell populations. To genetically label endothelial tip cells located at the vascular front, we generated double transgenic mice containing a *R26-tomato-EGFP* reporter^21^ in conjunction with a tamoxifen-inducible *iCre-ERT2* cassette under the control of the endothelial-specific molecule-1 (*Esm-1*) promoter^22^ (Fig. 3a). Previous studies showed *Esm-1* mRNA expression mainly in endothelial tip cells^23^,^24^. Accordingly, we observed specific expression of EGFP in endothelial cells at the vascular front 12 h after tamoxifen injection (Fig. 3b–d, bracket). To track labelled cells over time, we analysed the location of EGFP expressing cells within the vasculature after 24 or 48 h of tamoxifen injection (Fig. 3e–g, 48 h time point shown). We observed a striking bias of EGFP-positive cells towards the developing arteries, while we did not find a significant contribution of EGFP-positive cells to developing veins (Fig. 3g, arteries are marked by arrows, veins by arrowheads). After 48 h, about 15% of the arterial vessel area was occupied by EGFP-positive cells, while only about 1.5% of the venous vessel area was occupied by EGFP-positive cells (Fig. 3h). This suggests that similar to the vasculature in the regenerating zebrafish fin, endothelial tip cells in the mouse retina can contribute to nascent arteries, while they only marginally contribute to forming veins. EdU incorporation experiments in recombined endothelial cells and their descendants indicated proliferation of these cells (Supplementary Fig. 5), accounting for an increase in labelled endothelial cells at later time points.

### Expression of *cxcr4a* and its ligands *cxcl12a* and *cxcl12b*

**Figure 4:**
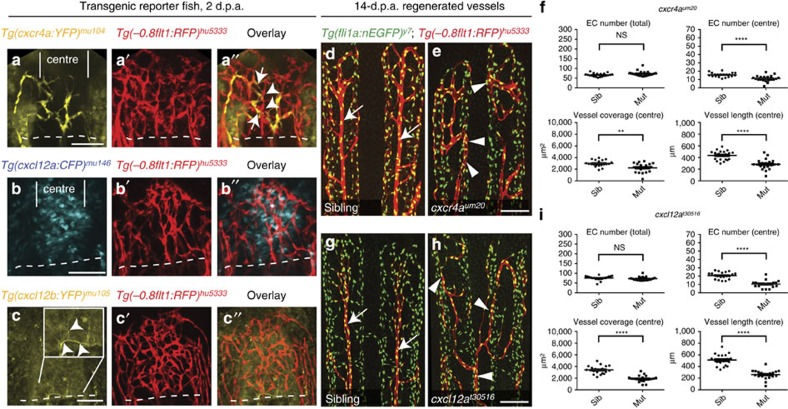
The chemokine receptor *cxcr4a* and its ligand *cxcl12a* are required for proper arterial patterning during fin regeneration. Dashed lines indicate amputation planes in **a**–**c**′′. (**a**) *Tg(cxcr4a:YFP)*^*mu104*^ fish reveal YFP expression in individual cells in the centre of the regenerating fin ray. (**a**′) *Tg(−0.8flt1:RFP)*^*hu5333*^ labelled blood vessels. (**a**′′) Overlay of red and yellow channels reveals expression of YFP in individual endothelial cells (arrows), while neighbouring endothelial cells do not express YFP (arrowheads). (**b**) CFP expression in the centre of the regenerating fin in *Tg(cxcl12a:CFP)*^*mu146*^ transgenic zebrafish. (**b**′) *Tg(−0.8flt1:RFP)*^*hu5333*^ labelled blood vessels. (**b**′′) Overlay of red and blue channels. (**c**) YFP expression in filamentous structures (arrowheads) extending into the regenerating fin in *Tg(cxcl12b:YFP)*^*mu105*^ transgenic zebrafish. (**c**′) *Tg(−0.8flt1:RFP)*^*hu5333*^ labelled blood vessels. (**c**′′) Overlay of red and yellow channels. (**d**) Fin vasculature in wild-type sibling 14 d.p.a. Arrows indicate *Tg(−0.8flt1:RFP)*^*hu5333*^-positive endothelial cells in the centre of the fin ray. (**e**) *cxcr4a*^*um20*^ mutant; arrowheads indicate ectopic *Tg(−0.8flt1:RFP)*^*hu5333*^-positive endothelial cells. (**f**) Quantification of artery formation defects in *cxcr4a*^*um20*^ mutants. Endothelial cell numbers, vessel coverage and length are reduced in the centre. (**g**) Fin vasculature in wild-type sibling 14 d.p.a. Arrows indicate *Tg(−0.8flt1:RFP)*^*hu5333*^-positive endothelial cells in the centre of the fin ray. (**h**) *cxcl12a*^*t30516*^ mutant; arrowheads indicate ectopic *Tg(−0.8flt1:RFP)*^*hu5333*^-positive endothelial cells. Scale bar (**a**–**c**,**e**,**h**), 100μm. (**i**) Quantification of artery formation defects in *cxcl12a*^*t30516*^ mutants. Endothelial cell numbers, vessel coverage and length are reduced in the centre. NS, not significant, **P<0.01, ****P<0.0001; Mann–Whitney *U*-test. Twenty individual fin rays from five fish were analysed.

We reasoned that pro-migratory genes specifically expressed by endothelial tip cells might control the observed distinct migratory behaviours of arterial-fated endothelial cells. We and others had previously shown that the chemokine receptor *Cxcr4* was specifically expressed in endothelial tip cells, both in mouse^23^,^24^ and zebrafish (*cxcr4a*)^15^. We therefore analysed expression of *cxcr4a* during fin regeneration. To achieve cellular resolution, we generated transgenic animals expressing fluorescent proteins under the control of the *cxcr4a*, *cxcl12a* and *cxcl12b* promoters^25^, respectively (see Methods). To visualize the vasculature in addition to transgene expression, we crossed these fish into the *Tg(−0.8flt1:RFP)*^*hu5333*^ background. In *Tg(cxcr4a:YFP)*^mu104^ zebrafish, we observed YFP expression mainly in the centre of regenerating fin rays, where it overlapped with individual endothelial cells in a salt and pepper distribution (Fig. 4a–a′′, compare cells marked by arrows and arrowheads). *Tg(cxcl12a:CFP)*^*mu146*^ fish showed expression of CFP in non-endothelial cells located in the centre of regenerating fin rays, in the area of the forming artery (Fig. 4b–b′′). Finally, in *Tg(cxcl12b:YFP)*^*mu105*^ fish, we observed YFP expression in filamentous structures, most probably nerve fibres (Fig. 4c–c′′)^26^. These staining patterns were mirrored by *in situ* hybridization results (Supplementary Fig. 6). Thus, mRNAs for the chemokine receptor Cxcr4a and for the ligands Cxcl12a and Cxcl12b are expressed in the regenerating zebrafish fin.

### Defects of *cxcr4a* and *cxcl12a* mutants in arterial patterning

To address the relevance of *cxcr4a* in endothelial cell migration, we analysed the regenerating vasculature in homozygous *cxcr4a*^*um20*^ mutant zebrafish^27^. Although the total endothelial cell numbers were not different between wild type and *cxcr4a*^*um20*^ mutant fish at any of the analysed time points, we detected a strong reduction in endothelial cell numbers, vascular coverage and vessel length in the centre of the fin rays in *cxcr4a*^*um20*^ mutants (Supplementary Fig. 7), suggesting that artery formation was severely impaired (Fig. 4d–f, 14 d.p.a. time point shown, Supplementary Tables 1 and 2). We also observed ectopically located *Tg(−0.8flt1:RFP)*^*hu5333*^*-*positive arterial cells in mutant fish, often running in parallel with veins (Fig. 4e, white arrowheads). We observed similar defects in arterial patterning in *cxcl12a*^*t30516*^ mutant zebrafish^28^ (Fig. 4g–i, 14 d.p.a. time point shown, Supplementary Fig. 8, Supplementary Tables 1 and 2). By contrast, *cxcl12b*^*mu100*^ mutant zebrafish^15^ did not show differences in arterial patterning when compared with wild-type siblings (Supplementary Fig. 9, Supplementary Tables 1 and 2). Despite these patterning defects, differentiation of arterial cells appeared unaffected, as we observed *Tg(−0.8flt1:RFP)*^*hu5333*^-positive endothelial cells in *cxcr4a*^*um20*^ mutants, albeit in ectopic locations. Thus, Cxcr4a-Cxcl12a signalling appears to be necessary for proper arterial morphogenesis, but not differentiation. Quantifying endothelial cell proliferation in *cxcr4a*^*um20*^ mutants (Supplementary Fig. 3c,d and Supplementary Movie 4), we detected a reduction in endothelial cell proliferation in both veins and arteries in these mutants by about 50% (Supplementary Fig. 3e). Therefore, while the endothelial cell migratory defects observed in *cxcr4a*^*um20*^ mutants specifically affected forming arteries, the proliferative defects were apparent both in arteries and veins.

### *cxcr4a* ^*um20*^ mutant endothelial cells show defective migration

**Figure 5:**
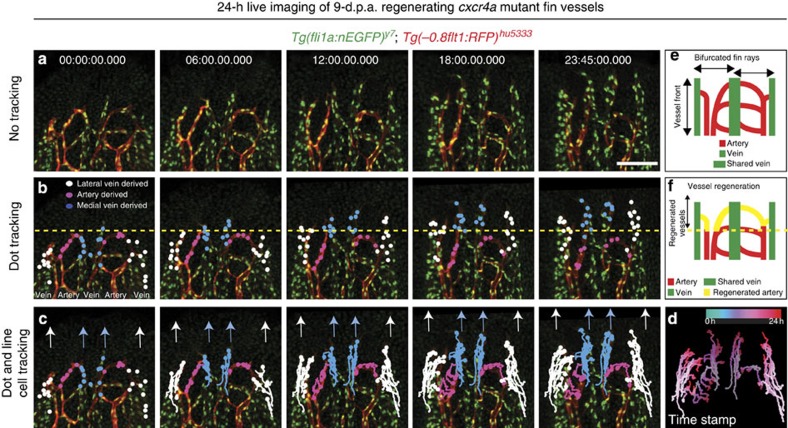
Time-lapse imaging of regenerating blood vessels in *cxcr4a*^*um20*^ mutant fish. (**a**) Still images taken every 6 h from a 24 h time-lapse movie on 9 d.p.a. fin regenerate of *cxcr4a*^*um20*^ fish. (**b**) Tracking of individual cells deriving form the lateral vein (white dots), the artery (pink dots) or the medial vein (blue dots). (**c**) Tracks of labelled cells in (**b**). White arrows indicate migration paths of lateral vein-derived endothelial cells, while blue arrows indicate migration paths of medial vein-derived endothelial cells. Note persistent migration of tracked endothelial cells in the direction of the growing regenerate. (**d**) Time stamp of tracked endothelial cells. (**e**) Schematic drawing of vessel front in the regenerated fin of *cxcr4a*^*um20*^ fish at 10 d.p.a. Arteries and veins are indicated. (**f**) Schematic drawing of *cxcr4a*^*um20*^ fin vasculature regenerated within 24 h from 9 d.p.a. Scale bar, 100 μm; d.p.a., days post amputation. Representative movie of a total of three movies.

To understand how Cxcr4a signalling influences artery formation, we performed time-lapse imaging of *cxcr4a*^*um20*^ mutant fish for 24 h, starting at 9 d.p.a. These studies revealed that in the absence of Cxcr4a signalling, endothelial tip cells failed to turn medially, and instead continued to migrate as a vascular front (Fig. 5a–c, time stamp in d, schematic drawings in e,f, Supplementary Movie 5, and compare with Fig. 2a–f). Consequently, no artery formed in the centre of the regenerating fin ray. By contrast, we observed *Tg(−0.8flt1:RFP)*^*hu5333*^-positive endothelial cells adjacent to the laterally located veins. Thus, Cxcr4a signalling is indispensible during the coordination of the complex endothelial cell movements that are necessary for artery formation during tissue regeneration.

### Cell transplantations reveal collective cell migration

**Figure 6:**
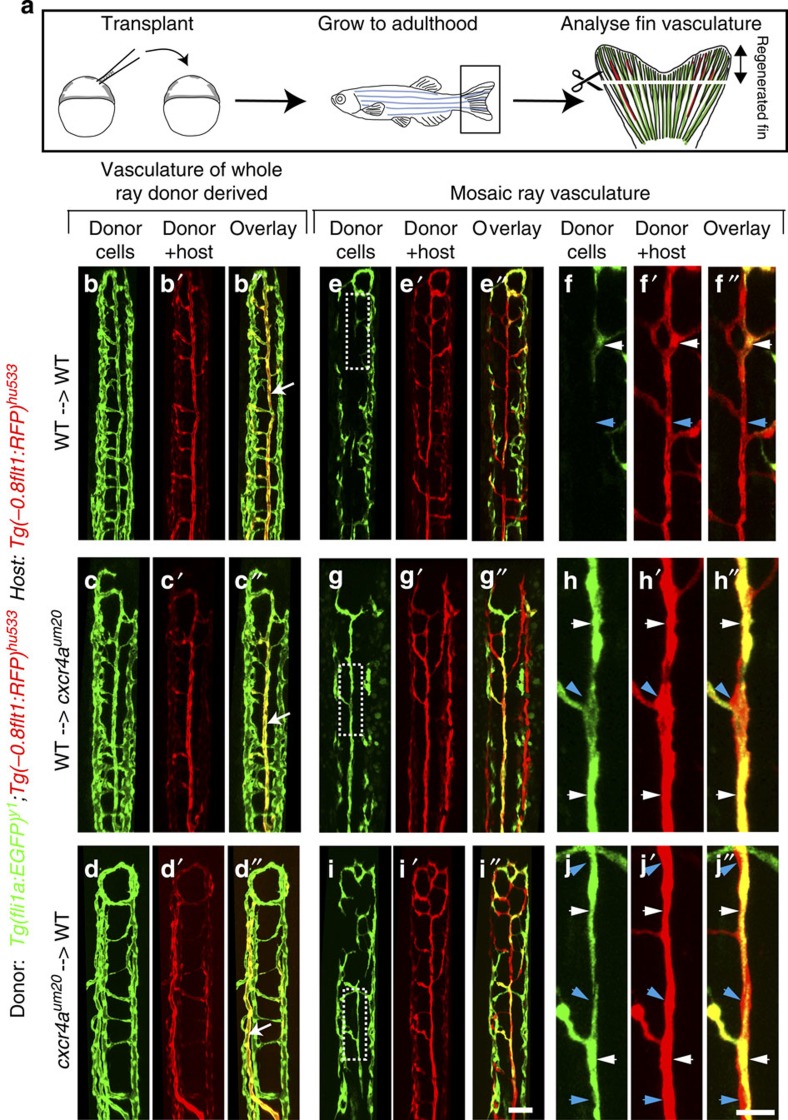
Analysis of *cxcr4a* function in chimeric fin vasculature. (**a**) Schematic drawing of transplantation procedure. Cells from double transgenic *Tg(fli1a:EGFP)*^*y1*^; *Tg(−0.8flt1:RFP)*^*hu5333*^ donors were transplanted into single *Tg(−0.8flt1:RFP)*^*hu5333*^ hosts. Two different scenarios were observed: Either the vasculature of the whole ray was donor-derived (**b**–**d**′′), or a mosaic ray vasculature, consisting of donor and host-derived endothelial cells formed (**e**–**j**′′). (**b**–**b**′′) In wild type to wild-type transplants, normal fin blood vessels formed. Arrow in **b**′′ marks artery (*n*=8 adult zebrafish). (**c**–**c**′′) When wild-type cells were transplanted into *cxcr4a*^*um20*^ mutant hosts, normal arteries could form (arrow in **c**″, *n*=2 adult zebrafish). (**d**–**d**′′) When *cxcr4a*^*um20*^ mutant cells were transplanted into wild-type hosts and formed the vasculature of an entire fin ray, arteries showed similar patterning defects as in *cxcr4a*^*um20*^ mutants (arrow in **d**′′, *n*=3 adult zebrafish). (**e**–**f**′′) In a control mosaic situation, both donor (white arrowhead, **f**–**f**′′) and host-derived (blue arrowhead, **f**–**f**′′) wild-type cells contributed to forming arteries. Dashed box in (**e**) indicates magnified area in **f**–**f**′′ (*n*=8 adult zebrafish). (**g**–**h**′′) In a mosaic situation, both donor-derived wild-type cells (white arrowheads in **h**–**h**′′) and host-derived *cxcr4a*^*um20*^ mutant cells (**h**–**h**′′, blue arrowheads) contributed to forming arteries. Dashed box in (**g**) indicates magnified area in **h**–**h**′′ (*n*=2 adult zebrafish). (**i**–**j**′′) In a mosaic situation, both donor-derived *cxcr4a*^*um20*^ mutant cells (white arrowheads in j–j’’) and host-derived wild-type cells (**j**–**j**′′, blue arrowheads) contributed to forming arteries. Dashed box in **i** indicates magnified area in **j**–**j**′′ (*n*=3 adult zebrafish). Scale bars are 50 μm in **i**′′ and 20 μm in **j**′′.

Migrating cells can either follow guidance cues individually or migrate as groups of cells^29^. Cxcr4 signalling in zebrafish has been described to play a role in single-cell migration regulating germ cell migration to the gonad and in collective cell migration during the formation of the lateral line^30^. To address whether Cxcr4a signalling within endothelial cells was necessary for single cell or collective cell migration, we generated chimeric embryos consisting of *cxcr4a*^*um20*^ mutant and wild-type cells and let them grow to adulthood. To visualize donor-derived endothelial cells, we transplanted double *Tg(fli1a:EGFP)*^*y1*^; *Tg(−0.8flt1:RFP)*^*hu5333*^ cells into single *Tg(−0.8flt1:RFP)*^*hu5333*^ hosts. We subsequently analysed fish that had donor- and host-derived blood vessels in their fins after amputation (Fig. 6a).

Of a total of about 5,200 transplanted embryos, 14 showed donor-derived cells in different adult fins (Supplementary Table 3). We observed two distinct patterns of donor cell contribution: either the vasculature of an entire given fin ray was donor-derived (Fig. 6b–d′′, arrows mark artery) or only a fraction of venous and/or arterial cells was donor-derived (Fig. 6e–j′′). In situations, where the entire fin ray vasculature was donor-derived, transplanted endothelial cells behaved according to the donor genotype. Wild-type cells transplanted to either wild-type hosts (Fig. 6b–b′′) or to *cxcr4a*^*um20*^ mutants (Fig. 6c–c″) formed normal arteries, while *cxcr4a*^*um20*^ mutant endothelial cells transplanted into wild-type hosts did not form arteries in the proper location (Fig. 6d–d′′). These observations suggest that *cxcr4a* functions endothelial cell autonomously during artery formation in regenerating fins.

In mosaic cases (Fig. 6e–j′′), we observed that donor (Fig. 6e,g,i,f–f′′, white arrowheads) and host cells (Fig. 6e′, blue arrowheads) could contribute to arteries, irrespective of their genotypes. These findings suggest that endothelial cells of the forming artery engage in collective migration, which allows for wild-type cells to rescue the migratory phenotype of *cxcr4a*^*um20*^ mutant cells. They furthermore suggest that, while showing migration defects, *cxcr4a*^*um20*^-deficient endothelial cells can properly differentiate into the arterial lineage.

### Ubiquitous overexpression of Cxcl12a rescues *cxcl12a* mutants

**Figure 7:**
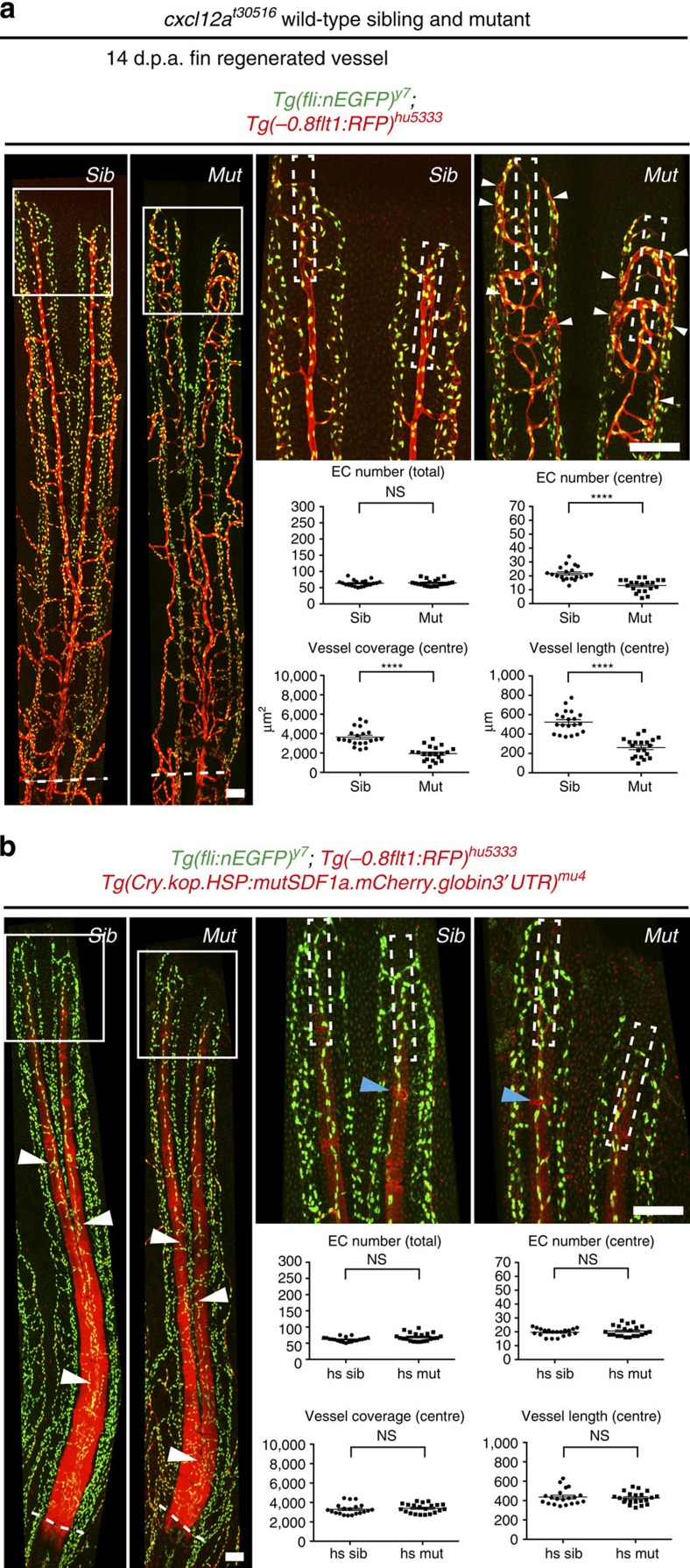
Global overexpression of Cxcl12a-mCherry rescues the vascular phenotype of *cxcl12*^*t30516*^ mutant fish. White dashed boxes indicate central areas quantified. Fin vasculature was analysed at 14 d.p.a. Dashed lines indicate amputation planes. (**a**) Heat-shocked control wild type and *cxcl12a*^*t30516*^ mutant fish not carrying the *Tg(Cry.kop.HSP:mutSDF1a.mCherry.globin3*′*UTR)*^*mu4*^ transgene. White arrowheads indicate ectopic *Tg(−0.8flt1:RFP)*^*hu5333*^-positive endothelial cells in *cxcl12a*^*t30516*^ mutants. Endothelial cell numbers, vessel coverage and vessel length are reduced in *cxcl12a*^*t30516*^ mutant fish. (**b**) Heat-shocked wild type and *cxcl12a*^*t30516*^ mutants carrying the *Tg(Cry.kop.HSP:mutSDF1a.mCherry.globin3*′*UTR)*^*mu4*^ transgene. None of the assayed parameters differs between heat-shocked wild type and *cxcl12a*^*t30516*^ mutants. Note accumulation of ubiquitously overexpressed Cxcl12a-mCherry protein in bony rays (white arrowheads) but not in the joints (blue arrowheads) between bone segments. ****P<0.0001; Mann–Whitney *U*-test, NS, not significant; *n*=8 adult zebrafish per stage. Scale bar, 100 um.

Our analysis of cxcl12a expression during fin regeneration (Fig. 4b–b′′) suggested that Cxcl12a might be distributed in a graded manner with higher concentrations in the centre of the fin. To determine whether graded Cxcl12a distribution was indeed necessary for proper artery formation, we ubiquitously overexpressed Cxcl12a-mCherry for 14 days during fin regeneration using *Tg(Cry.kop.HSP:mutSDF1a.mCherry.globin3*′*UTR)*^*mu4*^ fish. Overexpression of Cxcl12a-mCherry did not affect fin regeneration in wild-type siblings (Fig. 7, compare values for sibling in Fig. 7a with heat-shock sibling fish in Fig. 7b). Importantly, the arterial defects in *cxcl12a*^*t30516*^ mutant zebrafish were completely rescued by ubiquitous overexpression of Cxcl12a-mCherry (Fig. 7b, Supplementary Table 4). Surprisingly, we detected strong accumulation of the fusion protein in newly forming bone segments in the regenerates of both siblings and mutants (Fig. 7b, white arrowheads). We did not detect Cxcl12a-mCherry in the uninjured bone proximal to the amputation plane (Fig. 7b, proximal to the dotted line). The remainder of the regenerated fin, especially the most distal tissues, displayed faint Cxcl12a-mCherry signal, which accumulated in a dot-like pattern. Thus, although ubiquitously expressed under the control of a heat-shock promoter, Cxcl12a-mCherry accumulated in a tissue-restricted manner in regenerating fin tissue, with higher concentration detected within newly forming bones.

## Discussion

In this study, we have analysed endothelial cell migration during blood vessel plexus formation and remodelling in an *in vivo* setting: the regenerating zebrafish fin. Our results show that endothelial cells within growing blood vessel sprouts initially invade the avascular area. The signalling process that is most likely responsible for this behaviour is the VEGF pathway. During fin regeneration, *vegf-a* mRNA is being expressed in the fin regenerate and inhibition of VEGF signalling abolishes fin vessel outgrowth^31^. In addition, a VEGF gradient was postulated to drive retinal angiogenesis^32^. Studies in the mouse retina and in cell culture have shown that the exposure to VEGF can lead to the induction of tip cell specific genes, such as the Notch ligand *Dll4* and the chemokine receptor *Cxcr4* (refs 11, 23, 24, 33). Although activation of Notch signalling via Dll4 in neighbouring cells leads to the suppression of the tip cell phenotype^34^, Cxcr4a induction would change the tip cells’ responsiveness to guidance cues present in the environment. As we show that the chemokine ligand Cxcl12a is likely enriched in the central area of the fin ray, Cxcr4a expressing endothelial cells would respond to this chemokine and change their direction of migration towards the centre of the ray, allowing for proper arterial morphogenesis. Accordingly, our *cxcr4a*^*um20*^ mutant analysis revealed that in this setting, endothelial cells failed to turn medially, but continued to migrate in the direction of the avascular area. Therefore, Cxcr4a-mediated medial migration of tip cells would be necessary to balance the directionality imposed on the outgrowing vasculature by VEGF.

A dependence on Cxcr4 signalling for artery formation was recently observed in the mouse skin vasculature. In this setting, nerves secrete Cxcl12, while a subset of endothelial cells in the surrounding vascular plexus expresses Cxcr4 (ref. 35). In the absence of Cxcr4 signalling vessel nerve alignment is disrupted. This suggests that in the developing mouse skin vasculature, endothelial cell migration might occur similarly to what we observe in the regenerating zebrafish fin. In addition, Strasser *et al.*^24^ observed that, in retinae treated with the Cxcr4 inhibitor AMD3100, long sprouts formed that failed to connect laterally to neighbouring sprouts, a phenotype that corresponds to our observations during the live imaging of *cxcr4a*^*um20*^ mutants. This, together with our genetic lineage tracing of tip cells in the retina, suggests that the morphogenetic movements we observed during vascular plexus formation in the regenerating fin might be a general principle.

Despite the important role of Cxcr4a signalling during arterial morphogenesis, differentiation of arterial cells appears to be unaffected in *cxcr4a*^*um20*^ mutant zebrafish. We readily observe *Tg(−0.8flt1:RFP)*^*hu5333*^-positive blood vessels in *cxcr4a*^*um20*^ mutant fins, albeit in random locations. Proper arterial differentiation of endothelial cells lacking Cxcr4a function is furthermore supported by our cell transplantation experiments, where *cxcr4a*^*um20*^ mutant cells could contribute to arteries when surrounded by wild-type cells. This is in line with observations on Cxcr4-expressing endothelial cells from the skin of mouse embryos. In this setting, Cxcl12 exposure did not lead to arterial differentiation, while VEGF-A exposure could induce the expression of the arterial marker gene *ephrinB2* also in Cxcr4 mutant endothelial cells^35^. One important difference between the mouse skin and the regenerating fin vasculature is that, in the fin, arterial differentiation does not seem to depend on the proper location of the arterial-fated endothelial cells. In the mouse skin, only endothelial cells that are in close proximity to VEGF providing nerves can differentiate into arteries^7^,^36^. One reason for this difference might be that, during regeneration, tissue VEGF levels are high enough to allow for arterial differentiation throughout the entire fin.

Our data furthermore show that endothelial cell proliferation during vascular plexus formation differs between arterial and venous endothelial cells. We detect higher proliferation in venous endothelial cells. These findings are in agreement with previous studies in the mouse retina, which found persistent endothelial cell proliferation in veins, but not in arteries during vascular maturation^37^. Therefore, veins appear to constitute the main source of endothelial cells for newly forming blood vessels. In the mouse retina we find that descendants of previously labelled endothelial tip cells can proliferate during blood vessel formation and thereby account for an increase in the number of labelled endothelial cells. Our zebrafish data also show that both tip and stalk cells can proliferate during vascular outgrowth. This is in agreement with previous studies in developing zebrafish intersegmental vessels^38^,^39^,^40^. We observe a reduction in endothelial cell proliferation both in arteries and veins in *cxcr4a*^*um20*^ mutants. This is in contrast to the observed cell migration defects in *cxcr4a*^*um20*^ mutants, which specifically affect newly forming arteries. At present it is not clear whether the observed cell proliferation defects are due to a direct effect of *cxcr4a* signalling in both endothelial cell populations or due to perfusion defects, which might result from arterial malformations.

Chemokines play important roles in guiding the migration of various cell types, such as leukocytes^41^, endothelial cells^42^, endodermal cells^43^, germ cells^30^ and neurons^44^. They mainly function through the formation of a gradient, which provides positional information or through the activation of integrin signalling^30^. The zebrafish genome contains two *cxcl12* homologues^30^. In the embryo, *cxcl12b*^*mu100*^ mutants recapitulate the vascular phenotypes observed in *cxcr4a*^*um20*^ mutants^15^,^27^. Surprisingly, blood vessel formation during fin regeneration was unaffected in *cxcl12b*^*mu100*^ mutants, while *cxcl12a*^*t30516*^ mutants showed phenotypes similar to those of *cxcr4a*^*um20*^ mutants. It will be interesting in the future to determine the reason for this apparent switch in Cxcr4a ligand usage between embryonic stages and during regeneration.

The formation of chemokine gradients has been under intense investigation in recent years. One breakthrough study described the generation of chemokine gradients via ligand sequestration in the surrounding tissue by a decoy chemokine receptor, Cxcr7 (ref. 45). More recent reports show that migrating tissues can self-generate chemokine gradients^46^,^47^. We find selective accumulation of ubiquitously overexpressed Cxcl12a-mCherry protein in newly forming bones during zebrafish fin regeneration, which can fully rescue artery formation in *cxcl12a*^*t30516*^ mutants. This finding suggests that, while the precise mechanism is currently unknown, the regenerating fin tissue is either able to selectively degrade Cxcl12a protein outside of the bone or to stabilize it within the bone. Further studies will help to distinguish between these two options.

Our cell transplantation experiments showed that *cxcr4a*^*um20*^ mutant cells could contribute to the forming artery when surrounded by wild-type cells. This demonstrates that endothelial cells can also cooperate during plexus remodelling and that collective cell migration occurs during this process. Similar cell behaviours play a role during Cxcr4b controlled lateral line migration in zebrafish embryos^48^. In this setting, *cxcr4b*^*t26035*^ mutant cells can migrate normally in a wild-type primordium^49^. This indicates that, also in the vasculature, the organization of collective cell migration might be dependent on competition and cooperation between endothelial cells^29^.

Vascular malformations and patterning defects, including stenosis and arteriovenous shunts, can lead to life-threatening conditions in humans, underscoring the importance of understanding the mechanisms that lead to proper blood vessel sprouting and artery formation. Previous studies have used *in vitro* blood vessel sprouting assays to study endothelial cell dynamics during blood vessel growth. Arima *et al.*^14^ used an aortic ring sprouting assay and elegant cell tracking methods to show that endothelial cells in elongating branches are highly dynamic. The authors observed cell mixing and overtaking at the tip position. Interestingly, they also noticed individual endothelial cells that migrated opposite to the direction of the elongating sprout. The authors attributed this behaviour to loss of VEGF gradient formation in their culture setting. Our results now argue that this change in migratory direction could rather reflect the initiation of artery formation, not readily assessable in the aortic ring assay. In an embryoid body sprouting assay, Jakobsson *et al.*^13^ also observed endothelial cell mixing and shuffling of tip cells. These behaviours suggested competition between endothelial cells for the tip cell position, which was dependent on the relative levels of VEGF receptors and the Notch ligand Dll4. However, also in this assay, normal artery differentiation does not take place, precluding the analysis of the complete sprouting and remodelling programme. We now show that during zebrafish fin regeneration and in the developing mouse retinal vasculature, endothelial tip cells are derived from veins and that these tip cells can contribute to forming arteries. Thus, our results provide a new framework for an understanding of the coordinate migratory behaviours of endothelial cells that allow for the proper sprouting and subsequent remodelling of arteries and veins in a vascular plexus.

## Methods

### Zebrafish Strains and Fin amputations

Previously described zebrafish lines were *Tg(fli1a:EGFP)*^*y1*^ (ref. 18), (*Tg(fli1a:nEGFP)*^*y7*^ (ref. 20), Tg(−0.8*flt1:RFP)*^*hu5333*^ (ref. 19), *Tg(cxcl12b*^*BAC*^*:YFP)*^*mu105*^ (ref. 25), *cxcr4a*^*um20*^ (ref. 27), *cxcl12b*^*mu100*^ (ref. 15), *cxcl12a*^*t30516*^ (ref. 28). Zebrafish of 5–18 months of age were used. Fin amputations were performed as previously described^50^. All animal experiments were performed in compliance with the relevant laws and institutional guidelines and were approved by local animal ethics committees of the Landesamt für Natur, Umwelt und Verbraucherschutz Nordrhein-Westfalen.

### Generation of transgenic lines

We used Bacterial artificial chromosome (BAC) recombineering as described previously to generate transgenic lines^25^. To generate *Tg(cxcr4a*^*BAC*^*:YFP)*^*mu104*^ and *Tg(cxcl12a*^*BAC*^*:CFP)*^*mu146*^ animals, the start codon of the *cxcr4a* and *cxcl12a* gene in the BAC clone CH73-268G8 and CH73-353M13 was replaced with a Citrine or Cerulean cassette using Red/ET recombineering (GeneBridges). The Citrine cassette was amplified by PCR from pCS2+Citrine_kanR with the primers cxcr4a _HA1_GFP_fw (5′-ttattttttatttttacggctggtggggtagactttcgaga-aaatcggttACCATGGTGAGCAAGGGCGAGGAG-3′) and cxcr4a_HA2_kanR_rev (5′-gtgttgggctggggcgccgggggcttcagctcggtttccatcatgttataTCAGAAGAACTCGTCA-AGAAGGCG-3′). The Cerulean cassette was amplified by PCR from pCS2+Cerulean_kanR with the primers cxcl12a_HA1_gfp_fw (5′-cacagttgctcctggat-tctacacagtgcggatctcttcttcacactgcaAC-CATG-GTGAGCAAGGGCGAGGAG-3′) and cxcl12a_HA2_kanR_rev (5′-gcatgaatggcgaccgccatcagagcgactactacgatcactttgagatc-TCAGAAGAACTCGTCAAGAAGGCG -3′); homology to the BAC vector is depicted in lower case. *Tg(cxcl12b*^*BAC*^*:YFP)*^*mu105*^ was generated as previously described^25^. BAC DNA was isolated by Midiprep (Invitrogen) and injected at 100 pg per embryo together with 50 pg per embryo of *tol2* transposase mRNA into wild-type embryos.

The *Tg(Cry.kop.HSP:mutSDF1a.mCherry.globin3*′*UTR)*^*mu4*^ fish line was generated using the Tol2 transposon system. The transgene contains the HSP70/4 promoter (AF158020.1, GI:7108904) cloned upstream of the Danio rerio Cxcl12a coding sequence (FJ915063.1, GI:239596165) mutated in a way rendering it resistant for the morpholino oligonucleotide (ATGgacttgaaggtcatcg) and fused in frame to the mCherry open-reading frame (AB971706.1 GI:662033815). The Xenopus laevis globin3′UTR (J00978.1 GI:214209) was cloned downstream of the mCherry sequence.

### *In vivo* imaging of adult fish

The *in vivo* imaging of adult fish was carried out as described with the following modifications^51^. *Tg(fli1a:nEGFP*^*y7*^; −0.8*flt1:RFP*^*hu5333*^) fish were anaesthetized in 0.02% tricaine until they stopped swimming, transferred to the home-made chamber for live imaging, and immobilized by covering the trunk and caudal fin with 1% agarose. Afterwards, fish were orally administered with 126 mg l^−1^ tricaine in fish water at the speed of 5.5 ml min^−1^. We inserted a Silicone tubing (ID mm, OD 6 mm) into the fish mouth for tricaine delivery. The speed of tricaine delivery was controlled by a peristaltic pump (ISM-795C, ISMATEC). The removal of extra tricaine solution in the imaging chamber was achieved by connecting it to a fluid aspiration system for cell culture. Image acquisition was done for a maximum of 24 h every 15 min using a Leica SP5 confocal microscope equipped with a × 20 dipping lens. Confocal stacks and movies were assembled using Imaris software (Bitplane). The Linear Stack Alignment with SIFT plugin of ImageJ (NIH) was used for aligning image stacks. The MtrackJ plugin as implemented in ImageJ was used to label movies.

### Confocal microscopy and imaging processing

Fin regenerates were embedded in 1% low melting point agarose (Invitrogen) and imaged immediately after amputation. Images were acquired using a Leica SP5 confocal microscope and a Zeiss LSM780 confocal microscope. Maximum Projection images were generated with Imaris software (Bitplane). ImageJ (NIH) and Volocity 6.1 (PerkinElmer) were used to quantify cell number, vessel area and vessel length. We chose the third fin ray from the dorsal and/or ventral edge for our measurements. A region of interest (40 × 250 μm, width × height) in the centre of the growing vessel in the distal end of regenerating fin rays at 3 d.p.a. and two regions of interest of the same size in bifurcating fin rays from 5 d.p.a. onwards were measured. In total, the fin vasculature of at least ten fin rays from at least five fish was analysed for the each animal group of different regeneration stages. The Mann–Whitney non-parametric test as implemented in Prism 6.0b (Graphpad) was used to analyse the data.

### Quantification of endothelial cell proliferation in zebrafish fins by *in vivo* live imaging

Dividing endothelial cells were counted over 24 h in three independent movies each for wild-type and *cxcr4a*^um20^ mutants. Differences in proliferative behaviour between wild types and mutants were analysed in venous (lateral vein and medial vein combined) and arterial cell populations. The unpaired *t*-test as implemented in Prism 6.0b (Graphpad) was used for statistic analysis; significance (*P<0.05, **P<0.01) in unpaired *t*-test; NS=not significant.

### Heat-shock experiments

Heat shocks were carried out every 12 h on 14 consecutive days. For this purpose, fish were placed in system water, which was heated gradually for about 10–15 min from 28 °C to 37 °C in a standard waterbath. Subsequently, fish were exposed to 37 °C for 1 h, before gradual return to 28 °C.

### *In situ* hybridization

Whole-mount *in situ* hybridization was carried out as described^27^. Previously described probes were *cxcr4a*^27^, *cxcl12b*^27^ and cxcl12a^52^. Probes were generated from plasmid DNA. The plasmid containing *cxcr4a* was digested with NotI. The plasmids containing *cxcl12a* or *cxcl12b* were digested with NcoI. Sp6 was used to generate DIG-labelled antisense RNA for all three probes. DNA template for *in vitro* synthesis of *cxcr4a* sense probe was amplified from *pCRII-cxcr4a* by PCR. The forward primer 5′-GCTTGATTTAGGTGACACTATAGAATTATCGGGAACGGACTGG-3′ containing SP6 promoter and reverse primer 5′-AGGCGTACAGGATCGGG-3′ were used for PCR amplification. Proteinase K (30 μg ml^−1^) was used to permeabilize 2 d.p.a. fin regenerates for 20 min at room temperature before proceeding to the *in situ* hybridization with aforementioned probes. Images were acquired using a Leica M205 C Microscope and a Zeiss Axio Imager microscope.

### Blastomere transplantation

Cell transplantation was performed as described^40^. About 40 cells were transplanted from donor embryos to the margin of host embryos at sphere stage. The endothelial cell contribution of transplanted cells was assessed by visualization of EGFP and RFP expression. Adult fish containing EGFP and RFP expressing endothelial cells in the fin vasculature were selected for analysis.

### Genetic lineage tracing of endothelial tip cells

*Esm1(BAC)-iCreERT2* transgenics^22^ were bred into a *R26-tomato-EGFP*^21^ reporter background. Cre activity in newborn mice was induced by a single intraperitoneal injection of 50 μg 4-hydroxy tamoxifen solution (Sigma, H7904; 1 mg ml^−1^ in ethanol/peanut oil) at 12, 24 or 48 h before analysis at postnatal stage P6.

### Retina immunostaining for genetic lineage labelling

For retina staining, eyes were dissected and fixed in 4% PFA for 2 h at room temperature. Retinas were dissected, permeabilized and blocked in 1% BSA (Sigma, A4378-25G) and 0.3% Triton X-100 2 h at room temperature with agitation. Biotinylated isolectin B4 (Vector, B-1205, *Griffonia simlicifolia* lectin I; 1:25) in blocking buffer was added and incubated overnight at 4 °C with agitation. Next day, Retinas were washed three times in PBS and incubated with Alexa-Fluor-coupled streptavidin 647 (Invitrogen, 1:100) in blocking buffer together with GFP Alexa-Fluor-coupled 488 antibody (A21311, Invitrogen, 1:300) for 2 h at RT. Retinas were flat-mounted using Fluromount-G (SouthernBiotech, 0100-01) and images were taken with a Leica SP5 confocal microscope. Volocity software (PerkinElmer) was used for image processing and quantitative analysis.

### EdU labelling

Esm1-iCreERT2 ^+/T^, R26mTmG ^+/T^ double heterozygous mice were injected intragastric with 50 μl of 4-hydroxytamoxifen solution (0.25 mg ml^−1^; H7904 Sigma), at P2. Fifty microlitres of freshly prepared EdU nucleotides (2 mg ml^−1^ in PBS) was administered intraperitoneally at P6 and pups were dissected after 2 h.

### Whole-mount retina immunostaining and EdU detection

Retina immunostaining was performed according to Pitulescu *et al.*^53^ with some modifications. Whole eyes were fixed for 10 min at RT followed by 1 h on ice, with freshly prepared 4%PFA/PBS. Retinas were dissected and blocked/permeabilized (1% BSA, 0.3% Triton, PBS) for 30 min at RT. After rinsing twice with modified Pblec buffer (1 mM CaCl_2_, 1 mM MgCl_2_, 1 mM MnCl_2_, 0.4% Triton X-100 in PBS), retinas were incubated overnight at 4 °C with biotinylated *Griffonia simplicifolia* lectin I (isolectin B4) (1:50, VectorLabs, B-1205) and the following primary antibodies: rabbit anti-Erg (1:200, Santa-Cruz, sc-353) and chicken anti-GFP (1:300, Aves Labs, GFP-1010). On the next day, retinas were washed for 20 min in blocking buffer diluted once with PBS and three times (10 min per wash) in PBS. Retinas were then incubated for 2 h with CF^TM^405M-streptavidin antibody (1:100, Biotrend, 29033) or Alexa Fluor 546 streptavidin-conjugated (1:100, Invitrogen, S11225) and the following species-specific secondary antibodies: Alexa Fluor 488-donkey anti-chicken (1:500, Jackson ImmunoResearch Lab., 703-545-155), Alexa Fluor 647-goat anti-rabbit (1:500, Invitrogen, A21244) or Alexa Fluor 594-donkey anti-rabbit (1:500, Invitrogen, A21207). Retinas were then washed as described above. To detect EdU incorporation Click-IT EdU detection reactions were performed according to the manufacturer (Life Technologies, C103339 or C10340), using either Alexa Fluor 594 or 647 azide. Retinas were washed twice in PBS and flat-mounted on glass microscope slides using Fluoromount-G (Southern Biotech, 0100-01).

## Author contributions

C.X., R.H.A. and A.F.S. planned the experiments. C.X., I.S. and M.P. carried out the experiments. C.X., S.S.H., I.S., M.P., R.H.A. and A.F.S. analysed the data. S.F.R. generated the *esm1(BAC)-iCreERT2* transgenic mouse line. J.B. generated the *Tg(cxcr4a*^*BAC*^*:YFP)*^*mu104*^ transgenic zebrafish line. D.M. and E.R. contributed the *Tg(Cry.kop.HSP:mutSDF1a.mCherry.globin3*′*UTR)*^*mu4*^ fish line. C.X. and A.F.S. wrote the paper. All authors commented on the manuscript.

## Additional information

**How to cite this article:** Xu, C. *et al.* Arteries are formed by vein-derived endothelial tip cells. *Nat. Commun.* 5:5758 doi: 10.1038/ncomms6758 (2014).

## Supplementary Material

Supplementary Information Supplementary Figures 1-9 and Supplementary Tables 1-4.

Supplementary Movie 1 24 hour time-lapse movie of blood vessel growth in the regenerating wildtype zebrafish fin. Frames are taken every 15 minutes. *Tg(fli1a:nEGFP)^y7^* labels nuclei of all endothelial cells (green), *Tg(-0.8flt1:RFP)^hu5333^* labels arterial endothelial cells (red). Original movie is shown in addition to endothelial cell tracks. Pink dots label arterial endothelial cells, while white dots label endothelial cells derived from the lateral veins. Blue dots label endothelial cells in the medial vein.

Supplementary Movie 2 24 hour time-lapse movie of endothelial cell proliferation in the regenerating zebrafish fin. Frames are taken every 15 minutes. *Tg(fli1a:nEGFP)^y7^* labels nuclei of all endothelial cells (green), *Tg(-0.8flt1:RFP)^hu5333^* labels arterial endothelial cells (red). Proliferating endothelial cells are labeled by open and closed circles. Proliferating endothelial cells derived from arteries and lateral veins are labeled in red and yellow, respectively, while proliferating cells derived from medial vein are labeled in turquoise.

Supplementary Movie 3 4 hour time-lapse movie of tip and stalk cell proliferation in the regenerating fin of wild type fish. Frames are taken every 15 minutes. *Tg(fli1a:nEGFP)^y7^* labels nuclei of all endothelial cells (green), *Tg(-0.8flt1:RFP)^hu5333^* labels arterial endothelial cells (red). A proliferating endothelial tip cell is labeled in blue while two proliferating endothelial stalk cells are labeled in pink and white.

Supplementary Movie 4 24 hour time-lapse movie of endothelial cell proliferation in the regenerating zebrafish fin in a *cxcr4a^um20^* homozygous mutant. Frames are taken every 15 minutes. *Tg(fli1a:nEGFP)^y7^* labels nuclei of all endothelial cells (green), *Tg(-0.8flt1:RFP)^hu5333^* labels arterial endothelial cells (red). Proliferating endothelial cells are labeled by open and closed circles. Proliferating endothelial cells derived from arteries and lateral veins are labeled in red and yellow, respectively, while proliferating cells derived from medial veins are labeled in turquoise.

Supplementary Movie 5 24 hour time-lapse movie of blood vessel growth in the regenerating zebrafish fin in *cxcr4a^um20^* homozygous mutant. Frames are taken every 15 minutes. *Tg(fli1a:nEGFP)^y7^* labels nuclei of all endothelial cells (green), *Tg(-0.8flt1:RFP)^hu5333^* labels arterial endothelial cells (red). Original movie is shown in addition to endothelial cell tracks. Pink dots label arterial endothelial cells, while white dots label endothelial cells derived from the lateral veins. Blue dots label endothelial cells in the medial vein.
