# Supplementary material for: Arteries are formed by vein-derived endothelial tip cells
Source: Nat Commun. 2014 Dec 15;5:5758. doi: 10.1038/ncomms6758 (PMC4275597; doi:10.1038/ncomms6758)
Supplement: Supplementary Information — Supplementary Figures 1-9 and Supplementary Tables 1-4. [file ncomms6758-s1.pdf]

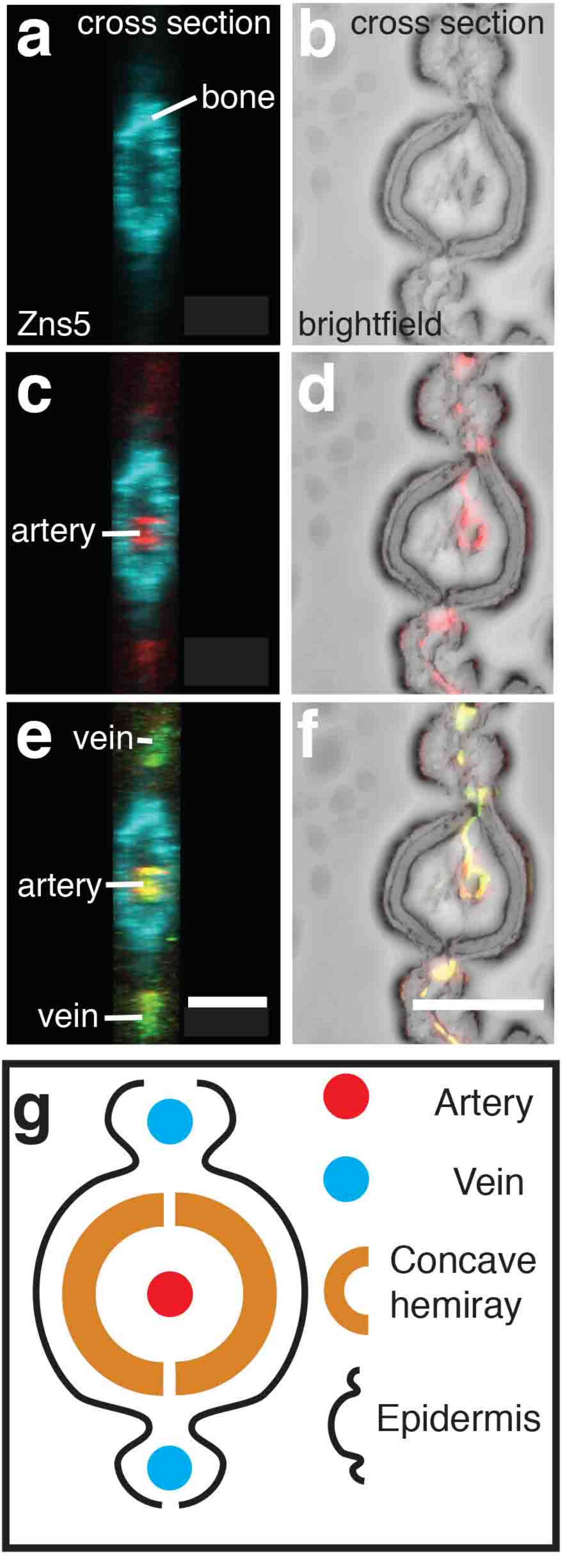

Supplementary Figure-1 Xu et al.

Supplementary Figure 1. Characterization of zebrafish fin vasculature. (a) Zns5 staining labelling the bone in a cross section of an individual fin ray. **(b)** Brightfield image of a cross section of an individual fin ray. **(c)** Overlay of *Tg(-0.8flt1:RFP)*^{hu5333} channel and Zns5 staining reveals location of the fin artery in the center of the bony ray. **(d)** Brightfield image of a cross section of an individual fin ray showing *Tg(-0.8flt1:RFP)*^{hu5333} channel. **(e)** Overlay of *Tg(-0.8flt1:RFP)*^{hu5333}*; Tg(fli1a:EGFP)*^{y1} channels and Zns5 staining reveals location of arteries and veins in respect to the fin ray bone. **(f)** Overlay of brightfield image with *Tg(-0.8flt1:RFP)*^{hu5333}*; Tg(fli1a:EGFP)*^{y1} channels. **(g)** Schematic drawing of fin tissue organization. Scale bars are 25um for e and 100um for f. Representative images of n = 5 adult zebrafish in 3 replicates are shown.

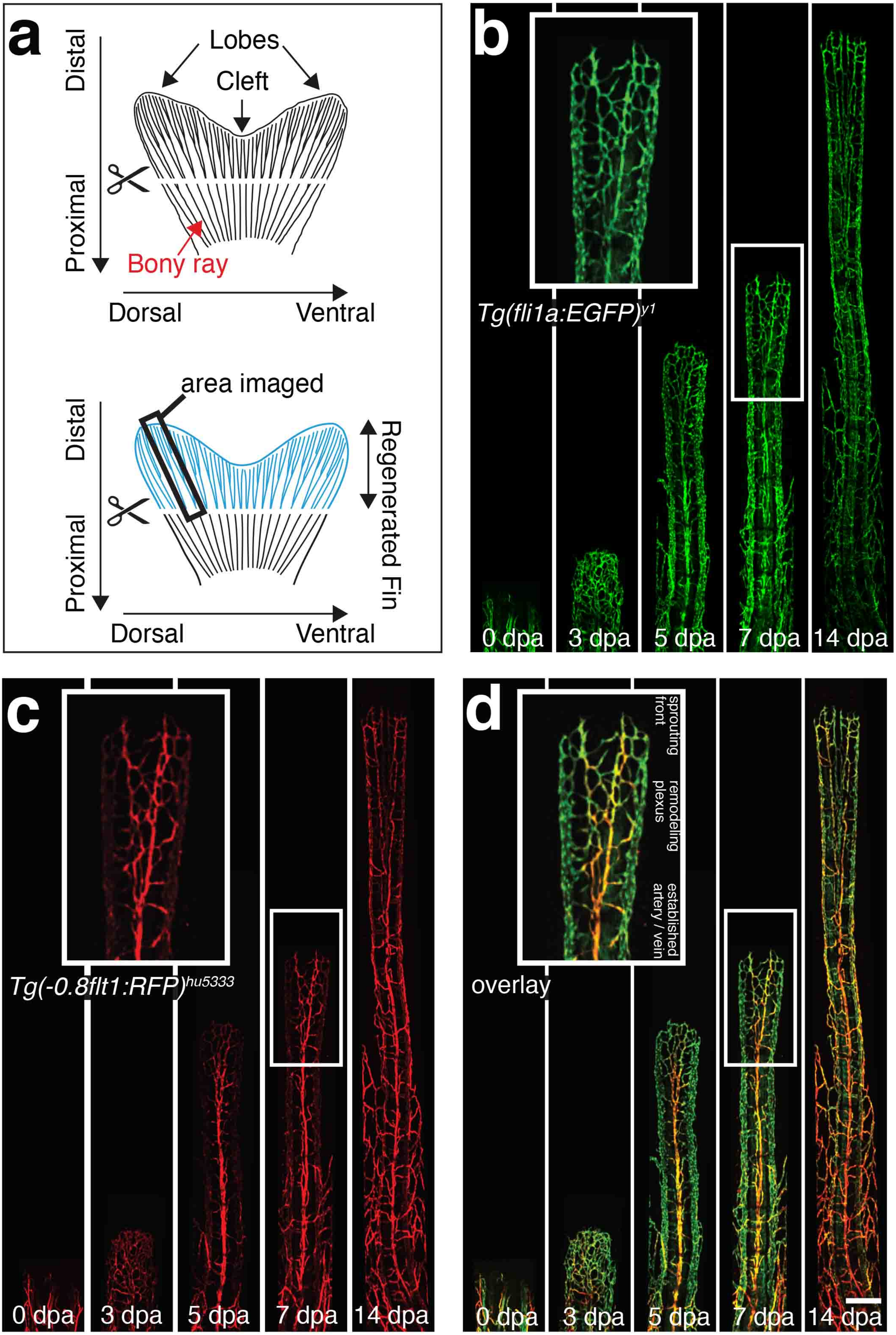

Supplementary Figure-2 Xu et al.

Supplementary Figure 2. Using transgenic zebrafish lines to label vasculature in the regenerated fin. (a) Schematic of zebrafish fin, indicating plane of amputation and imaged area. **(b)** *Tg(fli1a:EGFP)*^{y1} labels all blood vessels at the different stages of regeneration. **(c)** *Tg(-0.8flt1:RFP)*^{hu5333} preferentially labels arterial endothelial cells. **(d)** Overlay, highlighting the medially located arterial cells in yellow. Inset shows different areas of the outgrowing vessel. The remodeling vascular plexus is located proximal to the sprouting front and distally to the established arteries and vein. Scale bar is 200um. Representative images of n = 5 adult zebrafish in 3 replicates per stage are shown.

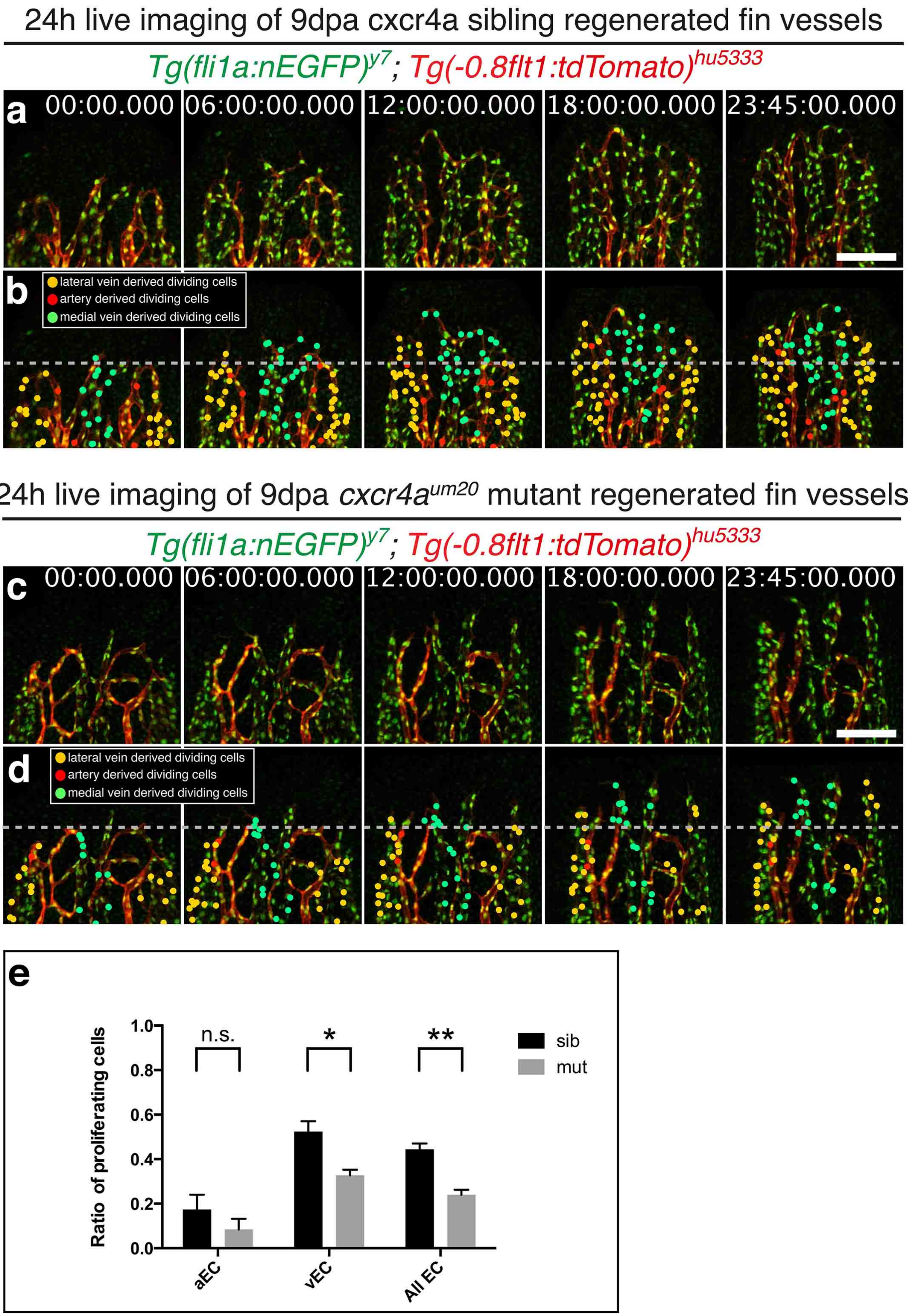

Supplementary Figure-3 Xu et al.

Supplementary Figure 3. Time-lapse imaging of regenerating blood vessels reveals differences in endothelial cell proliferation between arteries and veins and in *cxcr4a*^{um20} mutants. (**a**) Still images taken every 6 hours from a 24-hour time-lapse movie starting at 9 dpa in a wild type fish. (**b**) Tracking of individual dividing cells from lateral vein (yellow), artery (red) and medial vein (green) in wild type fish. (**c**) Still images taken every 6 hours from a 24-hour time-lapse movie starting at 9 dpa in a *cxcr4a*^{um20} mutant fish. (**d**) Tracking of individual dividing cells from lateral vein (yellow), artery (red) and medial vein (green) in a *cxcr4a*^{um20} mutant fish. (**e**) Quantification of proliferating arterial endothelial cells (aEC) and venous endothelial cells (vEC) in wildtype and *cxcr4a*^{um20} mutant fish. Significance (*$p < 0.05$, ** $p < 0.01$) in unpaired t-test with Welch's correction; n.s.: not significant. Scale bar is 100 um in (**a**) and (**c**). Representative still images of n = 3 adult wildtype and n = 3 *cxcr4a*^{um20} mutant zebrafish in 3 replicates each are shown.

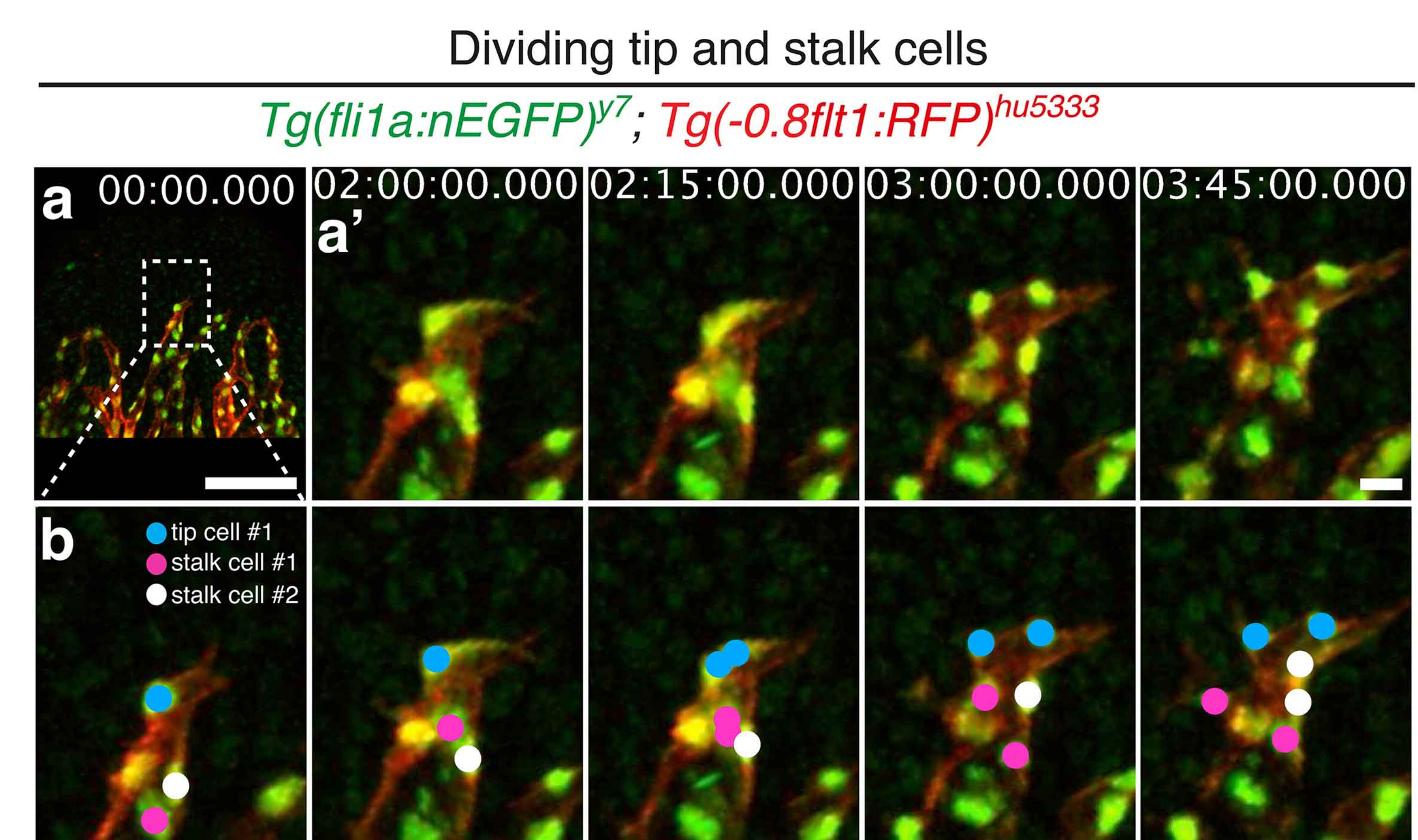
Dividing tip and stalk cells
Tg(fli1a:nEGFP)y7; Tg(-0.8flt1:RFP)hu5333
a
00:00.000
02:00:00.000
02:15:00.000
03:00:00.000
03:45:00.000
a’
b
tip cell #1
stalk cell #1
stalk cell #2

Supplementary Figure 4. Time-lapse imaging of proliferating tip and stalk cells in regenerating blood vessels of wild type fish. (**a**) Still images taken at the indicated time points from a 24-hour time-lapse movie starting at 9 dpa in wild type fish. (**a'**, **b**) Higher magnifications of selected area in (a). Proliferating tip cell (blue) and two stalk cells (pink and white) are marked. Scale bar is 100 um in a and 5 um in a'. Representative still images of n = 3 adult zebrafish in 3 replicates are shown.

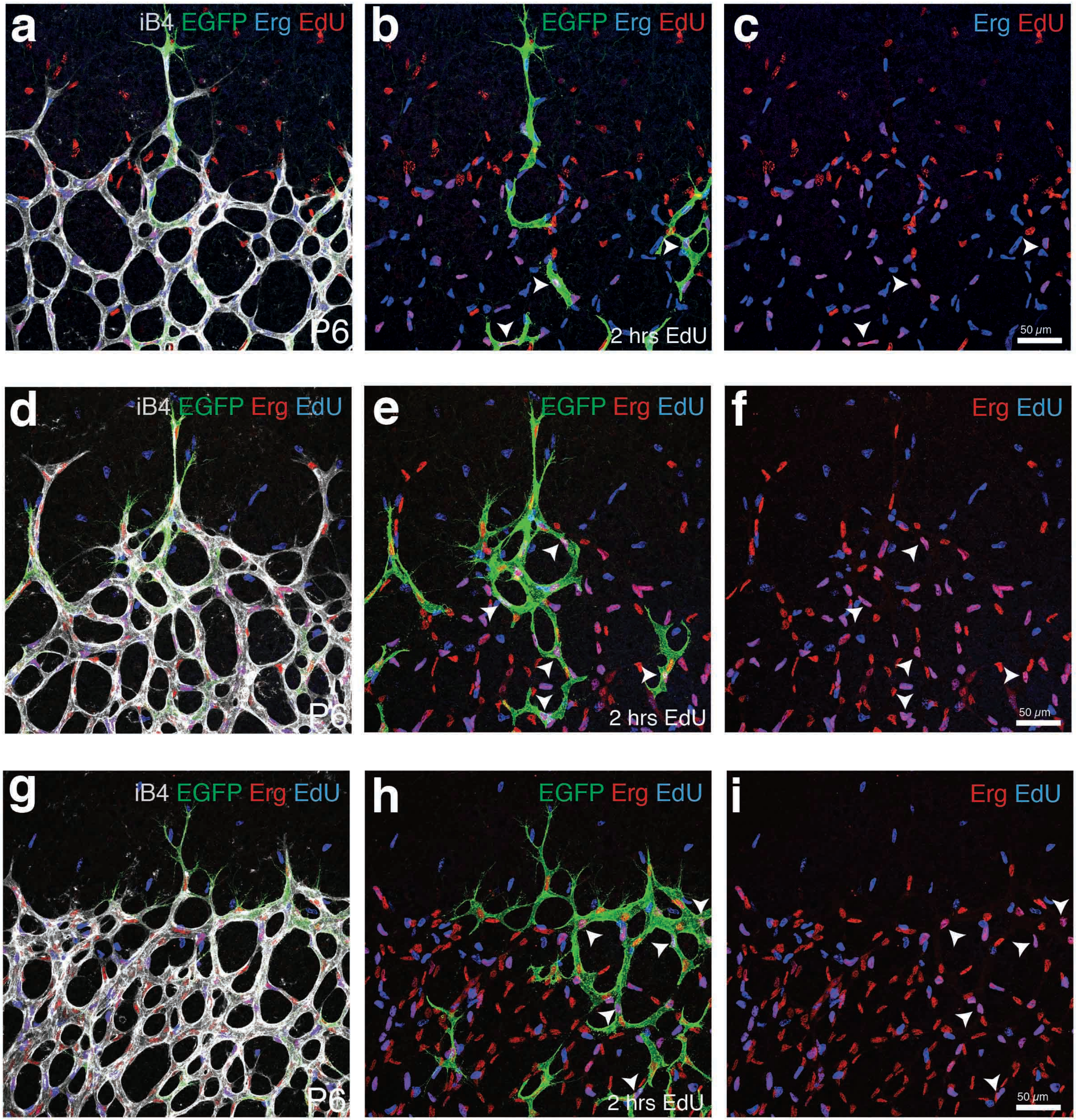

Supplementary Figure-5 Xu et al.

Supplementary Figure 5. Proliferation of genetically labeled Esm1-expressing descendant cells in retina. (**a-i**) Whole-mount retina immunostaining of EdU treated Esm1-iCreERT2 $^{+/T}$, R26mTmG $^{+/T}$ P6 mice. Mosaic gene deletion was induced at P2 with 4-hydroxy-tamoxifen. Arrowheads point to proliferating Esm1-expressing descendant cells. Representative immunofluorescence pictures were shown in (**a-c**) where stainings detect isolectin B4 (iB4, white, endothelial cells), EGFP (green), Erg (blue, endothelial cell nuclei), EdU (red) and in (**d-i**) isolectin B4 (iB4, white), EGFP (green), Erg (red), EdU (blue). Scale bar, 50 µm. (n=5)

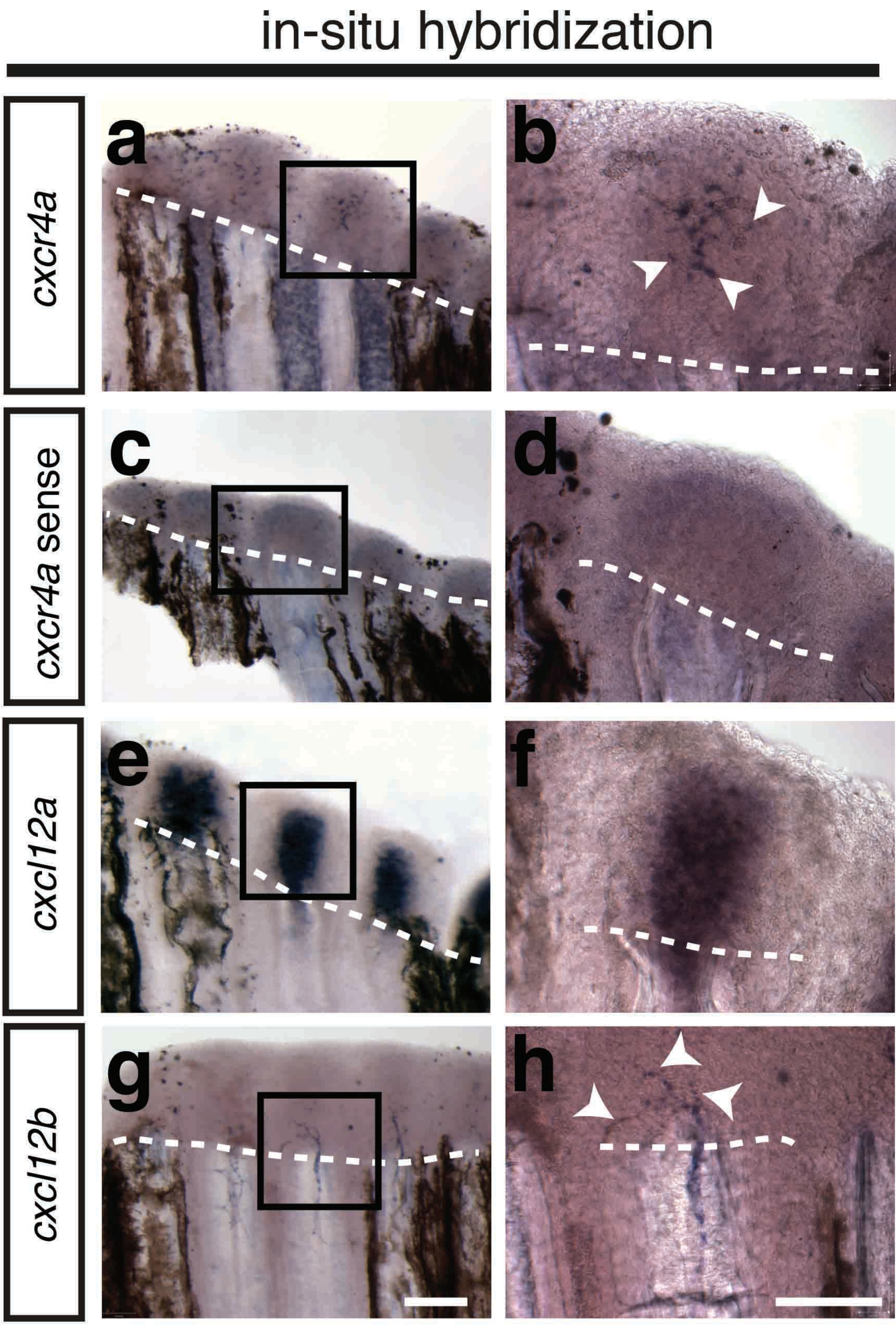

Supplementary Figure-6 Xu et al.

Supplementary Figure 6. Expression of the chemokine receptor *cxcr4a* and its ligands *cxcl12a* and *cxcl12b* during fin regeneration. (a-h) *in situ* hybridization on amputated fins with indicated probes. Boxes mark magnified areas. Dashed lines mark amputation planes. **(a, b)** *in situ* staining for *cxcr4a* reveals individual dots located in the center of the regenerating fin ray (arrowheads in b). **(c, d)** Sense probe control for *cxcr4a* shows no specific signal in the regenerating fin. **(e, f)** Staining for *cxcl12a* reveals strong staining in the center of the regenerating fin rays. **(g, h)** Expression of *cxcl12b* can be detected in filamentous structures located within the bony ray (arrowheads in h). Scale bars are 400um in (g) and 100um in (h). Representative images of n = 6 adult zebrafish in 3 replicates per staining are shown.

cxcr4a^{um20} wildtype sibling and mutant

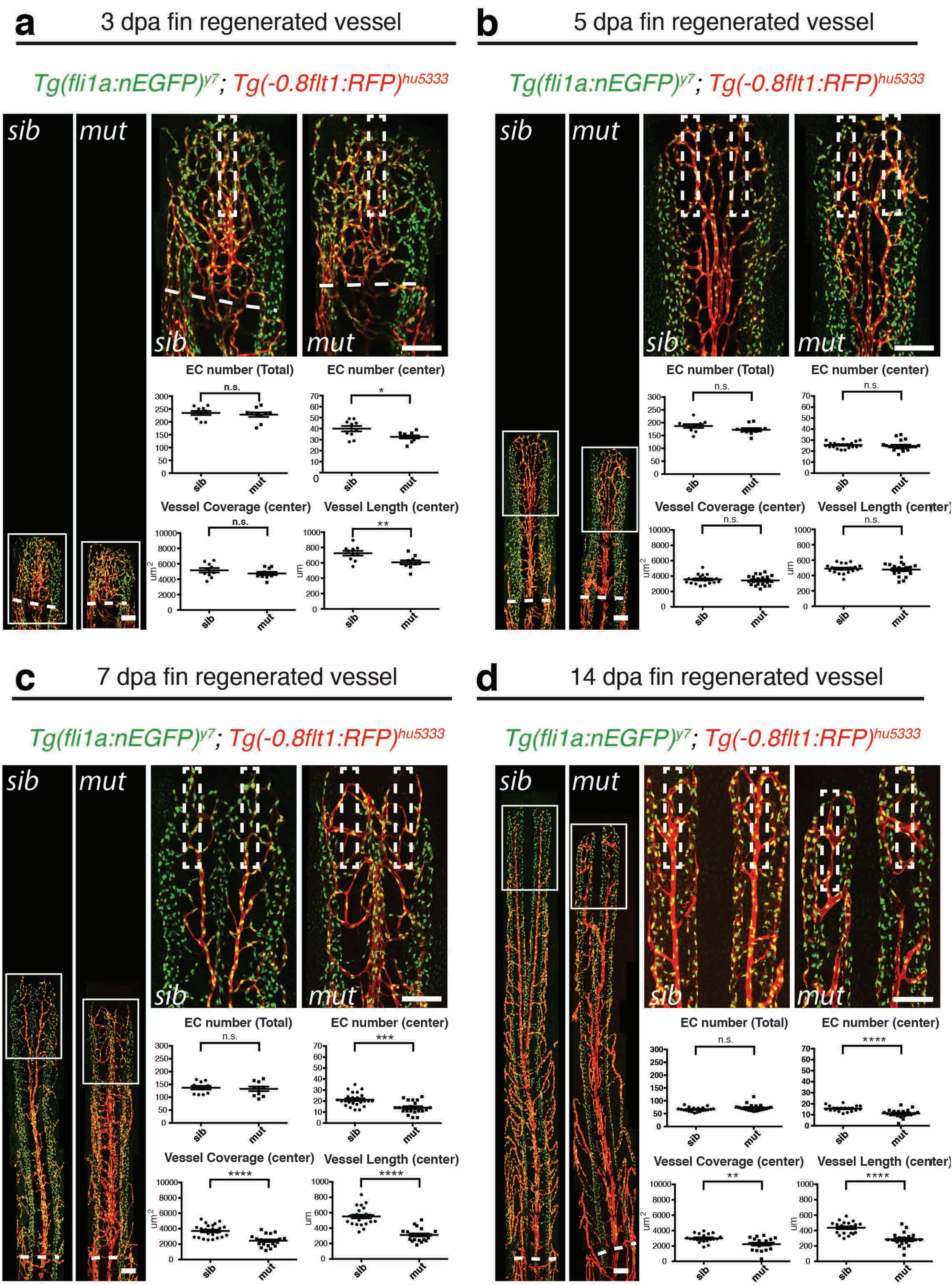

Supplementary Figure-7 Xu et al.

Supplementary Figure 7. Analysis of blood vessel remodeling in wildtype and *cxcr4a*^{um20} mutant zebrafish. White dashed boxes indicate central areas quantified. In "EC number (Total)" all endothelial cells in the image were counted. **(a)** 3 days post amputation. While total endothelial cell numbers are not different between wildtype and mutants, endothelial cell numbers in the center of the fin ray are lower, as is the length of the vessels in this area. Vessel coverage is unaffected. **(b)** 5 days post amputation. Neither of the analyzed parameters differs between wildtype and mutant fish. **(c)** 7 days post amputation. While total endothelial cell numbers are not different between wildtype and mutants, endothelial cell numbers in the center of the fin ray are lower. Vessel coverage and the length of the vessels in this area are also smaller. **(d)** 14 days post amputation. While total endothelial cell numbers are not different between wildtype and mutants, endothelial cell numbers in the center of the fin ray are lower. Vessel coverage and the length of the vessels in this area are also smaller. Scale bars are 100um. n.s. not significant, * $p<0.05$, ** $p<0.01$, *** $p<0.001$, ****, $p<0.0001$ (Mann-Whitney *U*-test). n = 8 adult zebrafish (3 replicates) per stage.

cxcl12a^{t30516} wildtype sibling and mutant

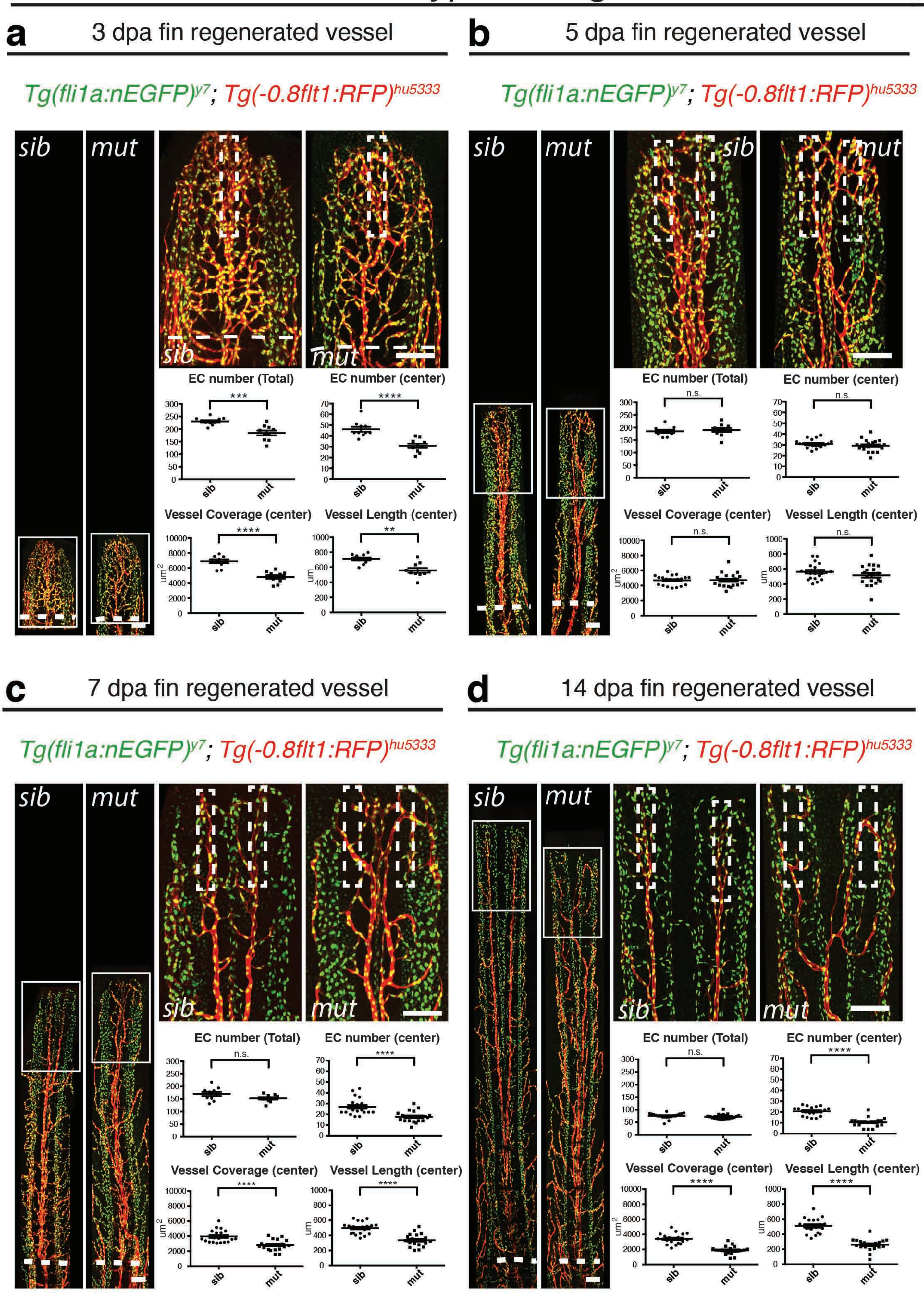

Supplementary Figure-8 Xu et al.

Supplementary Figure 8. Analysis of blood vessel remodeling in wildtype and *cxcl12a*^{t30516} mutant zebrafish. White dashed boxes indicate central areas quantified. In "EC number (Total)" all endothelial cells in the image were counted. **(a)** 3 days post amputation. Total endothelial cell numbers and endothelial cell numbers in the center of the fin ray are lower in mutants. Vessel coverage and the length of the vessels in this area are also smaller. **(b)** 5 days post amputation. Neither of the analyzed parameters differs between wildtype and mutant fish. **(c)** 7 days post amputation. While total endothelial cell numbers are not different between wildtype and mutants, endothelial cell numbers in the center of the fin ray are lower. Vessel coverage and the length of the vessels in this area are also smaller. **(d)** 14 days post amputation. While total endothelial cell numbers are not different between wildtype and mutants, endothelial cell numbers in the center of the fin ray are lower. Vessel coverage and the length of the vessels in this area are also smaller. Scale bars are 100um. n.s. not significant, * $p<0.05$, ** $p<0.01$, *** $p<0.001$, ****, $p<0.0001$ (Mann-Whitney *U*-test). n = 8 adult zebrafish (3 replicates) per stage.

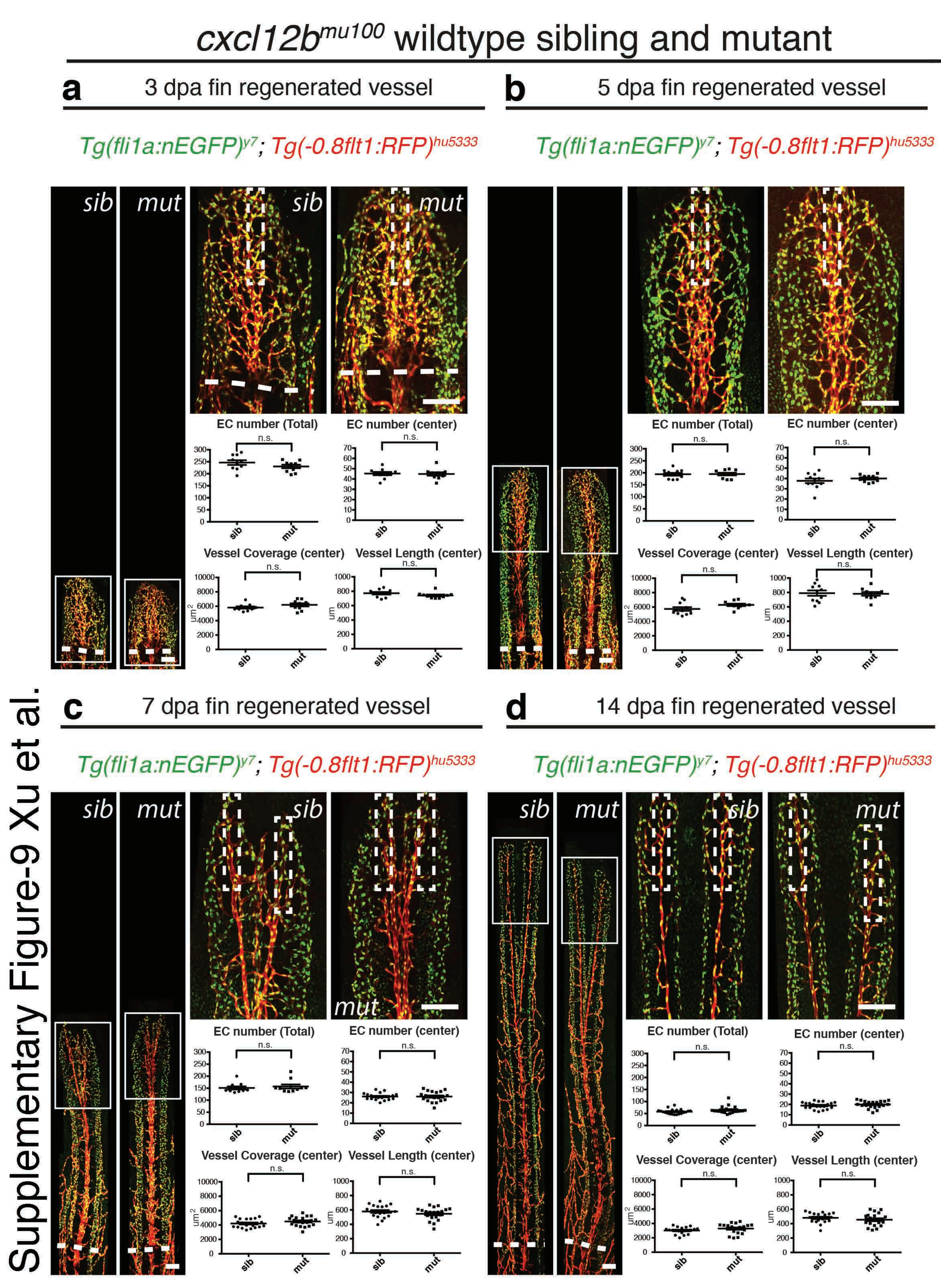

cxcl12b^mu100 wildtype sibling and mutant
a 3 dpa fin regenerated vessel
b 5 dpa fin regenerated vessel
c 7 dpa fin regenerated vessel
d 14 dpa fin regenerated vessel
Tg(fli1a:nEGFP)y7; Tg(-0.8flt1:RFP)hu5333
sib
mut
EC number (Total)
EC number (center)
Vessel Coverage (center)
Vessel Length (center)
n.s.
um2
um
Supplementary Figure-9 Xu et al.

Supplementary Figure 9. Analysis of blood vessel remodeling in wildtype and *cxcl12b*^{mu100} mutant zebrafish. White boxes indicate central areas quantified. In "EC number (Total)" all endothelial cells in the image were counted. Because bifurcation was delayed in both wildtype and mutant siblings in this line, we selected only one center at 5 dpa instead of 2 centers as for the other lines. None of the assayed parameters differs between wildtype and mutant fish. **(a)** 3 days post amputation. **(b)** 5 days post amputation. **(c)** 7 days post amputation. **(d)** 14 days post amputation. Scale bars are 100um. n.s. not significant, * $p<0.05$, ** $p<0.01$, *** $p<0.001$, ****, $p<0.0001$ (Mann-Whitney *U*-test). n = 8 adult zebrafish (3 replicates) per stage.

Supplementary Table 1. Quantification Summary of Endothelial Cell Number for ***cxcr4a, cxcl12a and cxcl12b*** Sibling and Mutant Animals

Quantification Summary of Endothelial Cell (EC) Number for *cxcr4a* Sibling and Mutant Animals

Reg. Stage	Sib EC number (Total)	Mut EC number (Total)	Mut-Sib Δ of EC number (Total)	%	Sig. Differ		Sib EC number (Center)	Mut EC number (Center)	Mut-Sib Δ of EC number (Center)	%	Sig. Differ
3 dpa	240	234	-6	-2	n.s.		42	33	-9	-22	*
5 dpa	191	169	-22	-11	n.s.		26	24	-2	-6	n.s
7 dpa	142	139	-4	-2	n.s.		20	13	-7	-35	***
14 dpa	66	70	4	6	n.s.		16	11	-5	-31	****

Quantification Summary of Endothelial Cell (EC) Number for *cxcl12a* Sibling and Mutant Animals

Reg. Stage	Sib EC number (Total)	Mut EC number (Total)	Mut-Sib Δ of EC number (Total)	%	Sig. Differ		Sib EC number (Center)	Mut EC number (Center)	Mut-Sib Δ of EC number (Center)	%	Sig. Differ
3 dpa	228	188	-40	-18	***		43	31	-13	-29	****
5 dpa	186	193	7	4	n.s.		30	29	-1	-3	n.s.
7 dpa	173	154	-19	-11	n.s.		24	18	-6	-25	****
14 dpa	77	71	-6	-8	n.s.		20	11	-9	-45	****

Quantification of Summary of Endothelial Cell (EC) Number for *cxcl12b* Sibling and Mutant Animals

Reg. Stage	Sib EC number (Total)	Mut EC number (Total)	Mut-Sib Δ of EC number (Total)	%	Sig. Differ		Sib EC number (Center)	Mut EC number (Center)	Mut-Sib Δ of EC number (Center)	%	Sig. Differ
3 dpa	247	239	-9	-3	n.s.		46	46	0	0	n.s.
5 dpa	191	195	4	2	n.s.		39	41	2	4	n.s.
7 dpa	142	150	9	6	n.s.		26	26	0	0	n.s.
14 dpa	57	61	4	7	n.s		19	20	1	5	n.s.

Reg. Stage: Regeneration Stage; Sig Differ: Significant Difference; n = 8 adult zebrafish (3 replicates) per stage. Mann-Whitney *U*-test.

n.s.	not significant
*	$p<0.05$
**	$p<0.01$
***	$p<0.001$
****	$p<0.0001$

Supplementary Table 2. Quantification Summary of Vessel Area and Vessel Length for ***cxcr4a, cxcl12a and cxcl12b*** Sibling and Mutant Animals

Quantification Summary of Vessel Area and Vessel Length for ***cxcr4a*** Sibling and Mutant Animals											
	Sib	Mut	Mut-Sib				Sib	Mut	Mut-Sib		
Reg. Stage	Vessel Area (μm^2)	Vessel Area (μm^2)	Δ of Vessel Area (μm^2)	%	Sig. Differ		Length (µm)	Length (µm)	Δ of Length (µm)	%	Sig. Differ
3 dpa	5068	4561	-507	-10	n.s.		738	595	-143	-19	**
5 dpa	3581	3323	-258	-7	n.s.		490	501	12	2	n.s
7 dpa	3810	2442	-1368	-36	****		551	305	-246	-45	****
14 dpa	2917	2293	-624	-21	**		459	281	-178	-39	****
Quantification Summary of Vessel Area and Vessel Length for ***cxcl12a*** Sibling and Mutant Animals											
	Sib	Mut	Mut-Sib				Sib	Mut	Mut-Sib		
Reg. Stage	Vessel Area (μm^2)	Vessel Area (μm^2)	Δ of Vessel Area (μm^2)	%	Sig. Differ		Length (µm)	Length (µm)	Δ of Length (µm)	%	Sig. Differ
3 dpa	7034	5046	-1988	-28	****		716	532	-184	-26	**
5 dpa	4847	4731	-116	-2	n.s.		525	547	23	4	n.s.
7 dpa	3689	2793	-896	-24	****		514	336	-178	-35	****
14 dpa	3394	1873	-1521	-45	****		506	268	-238	-47	****
Quantification Summary of Vessel Area and Vessel Length for ***cxcl12b*** Sibling and Mutant Animals											
	Sib	Mut	Mut-Sib				Sib	Mut	Mut-Sib		
Reg. Stage	Vessel Area (μm^2)	Vessel Area (μm^2)	Δ of Vessel Area (μm^2)	%	Sig. Differ		Length (µm)	Length (µm)	Δ of Length (µm)	%	Sig. Differ
3 dpa	5718	6336	618	11	n.s.		773	737	-36	-5	n.s.
5 dpa	5477	6236	759	14	n.s.		820	792	-28	-3	n.s.
7 dpa	4107	4522	415	10	n.s.		571	569	-2	0	n.s.
14 dpa	3073	3501	428	14	n.s		486	448	-38	-8	n.s.

Reg. Stage: Regeneration Stage; Sig Differ: Significant Difference; n = 8 adult zebrafish (3 replicates) per stage. Mann-Whitney *U*-test.

n.s.	not significant
*	$p<0.05$
**	$p<0.01$
***	$p<0.001$
****	$p<0.0001$

Supplementary Table 3. List of Transplanted Fish with Vasculature of Whole Fin Ray Donor Derived and Mosaic Ray Vasculature in The Ablated Fin and 14 dpa Regenerated Fin

Fish Number	Donor cell genotype	Host cell genotype	Type of fin	Fin vessels in the ablated fin			14dpa Regenerated fin vessels		
				Vasculature of whole fin Donor derived	Mosaic ray vasculature	Total number of analyzed fin rays	Vasculature of whole fin Donor derived	Mosaic ray vasculature	Total number of analyzed fin rays
No.1	WT Tg(fli1a:EGFP)	WT Tg(flt1:tdTomato)	Pectoral	* (3)	* (3)	6	* (3)	* (3)	6
No.2	WT Tg(fli1a:EGFP)	WT Tg(flt1:tdTomato)	Pectoral		* (1, artery)	1			
No.3	WT Tg(fli1a:EGFP; flt1:tdTomato)	WT Tg(flt1:tdTomato)	Caudal		* (1, vein)	1		* (1)	1
No.4	WT Tg(fli1a:EGFP; flt1:tdTomato)	WT Tg(flt1:tdTomato)	Pelvic		* (3)	3		* (2)	2
No.5	WT Tg(fli1a:EGFP; flt1:tdTomato)	WT Tg(flt1:tdTomato)	Pelvic	* (3)	* (3)	6	* (3)	* (2)	5
No.6	WT Tg(fli1a:EGFP)	WT Tg(flt1:tdTomato)	Caudal	* (4)	* (6)	10	* (6)	* (7)	13
No.7	WT Tg(fli1a:EGFP; flt1:tdTomato)	WT Tg(flt1:tdTomato)	Pectoral		* (1)	1		* (1)	1
No.8	WT Tg(fli1a:EGFP; flt1:tdTomato)	WT Tg(flt1:tdTomato)	Dorsal		* (2)	2		* (2)	2
No.9	*cxcr4a*^{um20} Tg(fli1a:EGFP; flt1:tdTomato)	WT Tg(flt1:tdTomato)	Pectoral		* (6)	6		* (8)	8
No.10	*cxcr4a*^{um20} Tg(fli1a:EGFP; flt1:tdTomato)	WT Tg(flt1:tdTomato)	Pectoral		* (9)	9		* (8)	8
No.11	*cxcr4a*^{um20} Tg(fli1a:EGFP; flt1:tdTomato)	WT Tg(flt1:tdTomato)	Caudal		* (4)	4		* (4)	4
No.12	WT Tg(fli1a:EGFP; flt1:tdTomato)	*cxcr4a*^{um20} Tg(flt1:tdTomato)	Dorsal	* (2)	* (2)	4	* (2)	* (2)	4
No.13	WT Tg(fli1a:EGFP; flt1:tdTomato)	*cxcr4a*^{um20} Tg(flt1:tdTomato)	Dorsal		* (1)	1		* (1)	1
				Total number of analyzed fin rays		54	Total number of analyzed fin rays		55

dpa: days post amputation
WT: wild type

n = 8 adult zebrafish (4 replicates) for WT to WT transplantation group highlighted in green in the table; n = 3 adult zebrafish (3 replicates) for *cxcr4a*^{um20} to WT transplantation group highlighted in yellow in the table; n = 2 adult zebrafish (2 replicates) for WT to *cxcr4a*^{um20} transplantation group highlighted in light blue in the table.

Supplementary Table 4

Quantification Summary of Endothelial Cell (EC) Number for ***cxcr12a*** Sibling and Mutant Animals

Reg. Stage	Sib	Mut	Mut-Sib				Sib	Mut	Mut-Sib		
	EC number (Total)	EC number (Total)	Δ of EC number (Total)	%	Sig. Differ		EC number (Center)	EC number (Center)	Δ of EC number (Center)	%	Sig. Differ
14 dpa	64	63	-1	-2	n.s.		21	15	-6	-29	****

Quantification Summary of Endothelial Cell (EC) Number for ***cxcl12a*** Sibling and Mutant Animals Overexpressing Cxcl12a

Reg. Stage	Sib	Mut	Mut-Sib	%	Sig. Differ		Sib	Mut	Mut-Sib	%	Sig. Differ
	EC number (Total)	EC number (Total)	Δ of EC number (Total)	%	Sig. Differ		EC number (Center)	EC number (Center)	Δ of EC number (Center)	%	Sig. Differ
14 dpa	61	66	5	8	n.s.		20	20	0	0	n.s.

Quantification Summary of Vessel Area and Vessel Length for ***cxcr4a*** Sibling and Mutant Animals

Reg. Stage	Sib	Mut	Mut-Sib				Sib	Mut	Mut-Sib		
	Vessel Area (µm^2)	Vessel Area (µm^2)	Δ of Vessel Area (µm^2)	%	Sig. Differ		Length (µm)	Length (µm)	Δ of Length (µm)	%	Sig. Differ
14 dpa	3384	1912	-1472	-43	****		500	281	-219	-44	****

Quantification Summary of Vessel Area and Vessel Length for ***cxcl12a*** Sibling and Mutant Animals Overexpressing Cxcl12a

Reg. Stage	Sib	Mut	Mut-Sib	%	Sig. Differ		Sib	Mut	Mut-Sib	%	Sig. Differ
	Vessel Area (µm^2)	Vessel Area (µm^2)	Δ of Vessel Area (µm^2)	%	Sig. Differ		Length (µm)	Length (µm)	Δ of Length (µm)	%	Sig. Differ
14 dpa	3182	3343	161	5	n.s.		420	426	6	1	n.s.

Reg. Stage: Regeneration Stage; Sig Differ: Significant Difference; n = 8 adult zebrafish (3 replicates) per stage. Mann-Whitney *U*-test.

n.s.	not significant
*	$p<0.05$
**	$p<0.01$
***	$p<0.001$
****	$p<0.0001$
